# Environmental heterogeneity shapes physiological traits in tropical direct‐developing frogs

**DOI:** 10.1002/ece3.7521

**Published:** 2021-05-01

**Authors:** Ruth Percino‐Daniel, José M. Contreras López, Oswaldo Téllez‐Valdés, Fausto R. Méndez de la Cruz, Alejandro Gonzalez‐Voyer, Daniel Piñero

**Affiliations:** ^1^ Departamento de Ecología Evolutiva Instituto de Ecología Universidad Nacional Autónoma de México Mexico City Mexico; ^2^ Posgrado en Ciencias Biológicas Universidad Nacional Autónoma de México Mexico City Mexico; ^3^ Instituto de Ciencias Biológicas Universidad de Ciencias y Artes de Chiapas Tuxtla Gutiérrez Mexico; ^4^ Facultad de Estudios Superiores Unidad de Biotecnología y Prototipos (UBIPRO) Iztacala Tlalnepantla Mexico; ^5^ Departamento de Zoología Laboratorio de Herpetología Instituto de Biología Universidad Nacional Autónoma de México Mexico City Mexico

**Keywords:** acclimatization, amphibians, critical thermal limits, elevation gradient, thermal ecology, vulnerability, warming tolerance

## Abstract

1. Tropical ectotherm species tend to have narrower physiological limits than species from temperate areas. As a consequence, tropical species are considered highly vulnerable to climate change since minor temperature increases can push them beyond their physiological thermal tolerance. Differences in physiological tolerances can also be seen at finer evolutionary scales, such as among populations of ectotherm species along elevation gradients, highlighting the physiological sensitivity of such organisms.
2. Here, we analyze the influence of elevation and bioclimatic domains, defined by temperature and precipitation, on thermal sensitivities of a terrestrial direct‐developing frog (*Craugastor loki)* in a tropical gradient. We address the following questions: (a) Does preferred temperature vary with elevation and among bioclimatic domains? (b) Do thermal tolerance limits, that is, critical thermal maximum and critical thermal minimum vary with elevation and bioclimatic domains? and (c) Are populations from high elevations more vulnerable to climate warming?
3. We found that along an elevation gradient body temperature decreases as environmental temperature increases. The preferred temperature tends to moderately increase with elevation within the sampled bioclimatic domains. Our results indicate that the ideal thermal landscape for this species is located at midelevations, where the thermal accuracy (*d_b_*) and thermal quality of the environment (*d_e_*) are suitable. The critical thermal maximum is variable across elevations and among the bioclimatic domains, decreasing as elevation increases. Conversely, the critical thermal minimum is not as variable as the critical thermal maximum.
4. Populations from the lowlands may be more vulnerable to future increases in temperature. We highlight that the critical thermal maximum is related to high temperatures exhibited across the elevation gradient and within each bioclimatic domain; therefore, it is a response to high environmental temperatures.

## Graphical abstract

Our study shows how the physiological thermal traits on a finer scale are shaped by heterogeneity environmental of the frogs of genus *Craugastor*. We measured different ecophysiological thermal traits in 15 populations along an elevation gradient and five bioclimatic landscapes and estimated the warming tolerance and cooling tolerance of the frogs. We highlight that the critical thermal limit maximum is a response to high environmental temperatures, suggesting the role of thermal plasticity is expressed in response to variation in environmental temperature. The results contribute to the understanding how resilience is critical in a warming world.

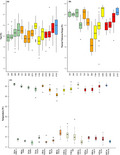

## 1 INTRODUCTION

A challenge for ecology and evolutionary biology is understanding how different biotic and abiotic environmental factors can influence the phenotype and genome expression (Chown et al., 2004). Temperature plays a key role affecting organisms as it influences nearly all biological processes at different levels of biological organization, from molecular kinetics to macroevolutionary rates of diversification (Dugo‐Cota et al., 2015; Kingsolver, 2009). In ectothermic organisms, environmental temperature is particularly important because it not only determines body temperature but also influences a variety of processes such as periods of activity, metabolism, locomotion, foraging ability, and courtship (Angilletta et al., 2002). Therefore, temperature is not merely an abiotic factor for ectotherms, but could be considered as part of the biotic environment (Angilletta, 2009).

Body temperature in ectotherms varies with environmental conditions (Hillman et al., 2008), making thermal sensitivities also important since they relate to the way in which organism performance depends on temperature (Angilletta, 2009). Amphibians differ from other vertebrate ectotherms in the strength with which temperature and water balance affects performance (Navas et al., 2008). Additionally, the interplay between these factors is dependent on a variety of conditions such as natural history, microhabitat, latitude, and elevation (Wells, 2007).

Tropical ectotherm species have been hypothesized to have narrow physiological limits compared with temperate species (Janzen, 1967). This is known as the climate variability hypothesis (Ghalambor, 2006), which has been tested at macroecological scales in a variety of organisms (Chown et al., 2004; Gunderson & Stillman, 2015). However, at finer scales our knowledge is still limited (Montejo‐Kovacevich et al., 2020). This limitation is particularly important for understanding the role of plasticity on thermal tolerance or species physiological limits. Therefore, there is some urgency to generate a better understanding of the role of thermal tolerance given that differences in physiological tolerances can also be seen at finer evolutionary scales. Such knowledge can be key to predicting species responses to climate change and species vulnerability, particularly in amphibians (Duarte et al., 2012).

In a landscape with an elevation gradient, climatic conditions vary with altitude; temperatures decrease as elevation increases, and precipitation regimes vary and are influenced by the orientation of the elevation gradient. These environmental variables grouped together in geographical space compose bioclimatic domains (Londoño‐Murcia et al., 2010). Therefore, the physiological traits of organisms along the gradient are predicted to show elevated plasticity to adjust to varying environment conditions. Such physiological plasticity to environmental conditions is called thermal acclimatization, which can be defined as a reversible change in a biological trait in response to temperature variability (Gunderson & Stillman, 2015). Environmental variability includes changes in temperature or humidity due to natural fluctuations encountered along environmental gradients or different seasons (Angilletta, 2009). In other words, acclimatization refers to the plasticity in thermal physiology expressed in response to predictable or stochastic environmental fluctuations like those encountered along an elevation gradient. Moreover, ectotherms also have thermoregulation strategies to compensate for extreme environmental conditions (Muñoz & Losos, 2018) that include behavioral responses such as microhabitat selection, allowing finer modulation of body temperature in some amphibian species (Farallo et al., 2018). Nonetheless, most amphibians are considered to be thermoconformers in that their body temperature is similar to the environmental temperature. Thus, the interplay between physiology and behavior may have an important influence on how some amphibians navigate the thermal landscape (Angilletta et al., 2002).

One approach to analyze how temperature influences organism performance, in addition to field body temperature, is to study the critical thermal limits of a species to understand physiological limitations to an organism's response to environmental heterogeneity. Critical thermal limits include the upper thermal limit or critical thermal maximum (*CT_max_*) and lower thermal limit or critical thermal minimum (*CT_min_*). Results in the literature are mixed regarding variation in critical thermal limits, with some studies suggesting that critical thermal limits decrease with elevation (von May et al., 2017), as predicted by theory, while others do not find any association (Christian et al., 2008). The main conclusion therefore seems to be that the response depends on the studied clade (von May et al., 2017). A study on toads within the genus *Rhinella* found that two species are strong thermoconformers, being highly dependent on environmental temperature and their thermal limits are not variable with respect to seasonal variation of climatic parameters (Anderson et al., 2018). On the other hand, for two species of frogs within the genus *Craugastor* in Costa Rica, the critical thermal limits are associated with the habitat the species occupies. *CT_max_* was found to be higher in the species that is more tolerant to deforestation and warming temperatures (*C. fitzingeri*), while the other (*C. crassidigitus*) is less tolerant to fragmentation and deforestation and presents lower critical thermal limits (Frishkoff et al., 2015). Studies on some ecthotherms show variation in upper and lower thermal limits associated with the species environment (Hoffmann et al., 2013). In light of global warming, the critical thermal limits are often considered as a proxy for vulnerability to climate change (Seebacher et al., 2015; Sinervo, 2011). It is important to consider life history, behavior, and the scaling of climate data to assess vulnerability to climate change (Nadeau et al., 2017). Also, the evolutionary potential to face global warming is equally important to understand the response to novel selective pressures (Muñoz et al., 2014).

Direct‐developing frogs are among the most diverse group of frogs in the New World (Frost, 2020), with over 900 species the group represents ca. 1/3 of Neotropical amphibian diversity and 120 species of this group mainly occur in Middle America. Many species are also polytypic, with striking variation in color patterns and are primarily characterized by the lack of a water‐dwelling larval phase and their use of substrate humidity for reproduction (Duellman & Trueb, 1994). *Craugastor loki* is a direct‐developing frog species found in North and Central America from southern Mexico to northern El Salvador, inhabiting both dry and humid habitats. This species is often locally abundant and has a broad elevational distribution from sea level to 2,200 m a.s.l., particularly in southern Mexico (Lynch, 2000), but also in Guatemala and El Salvador. Consequently, this species is particularly suitable for exploring how thermal sensitivities can vary at the local scale due to plasticity or thermal adaptation. Here, we analyze whether thermal sensitivities of *C. loki* are associated with elevation and bioclimatic domains, defined as an environmental space that integrates bioclimatic variables of temperature and precipitation. We addressed the following questions: (a) does temperature preference vary according to altitude and bioclimatic domains; (b) are thermal limits, *CT_max_* and *CT_min_*, lower as elevation increases as predicted by the climate variability hypothesis; (c) do thermal limits change in response to local variation in average maximum and minimum temperatures; and (d) are populations from high elevations more vulnerable to a warming climate?

## 2 MATERIALS AND METHODS

### 2.1 Study area and sampling

**FIGURE 1:**
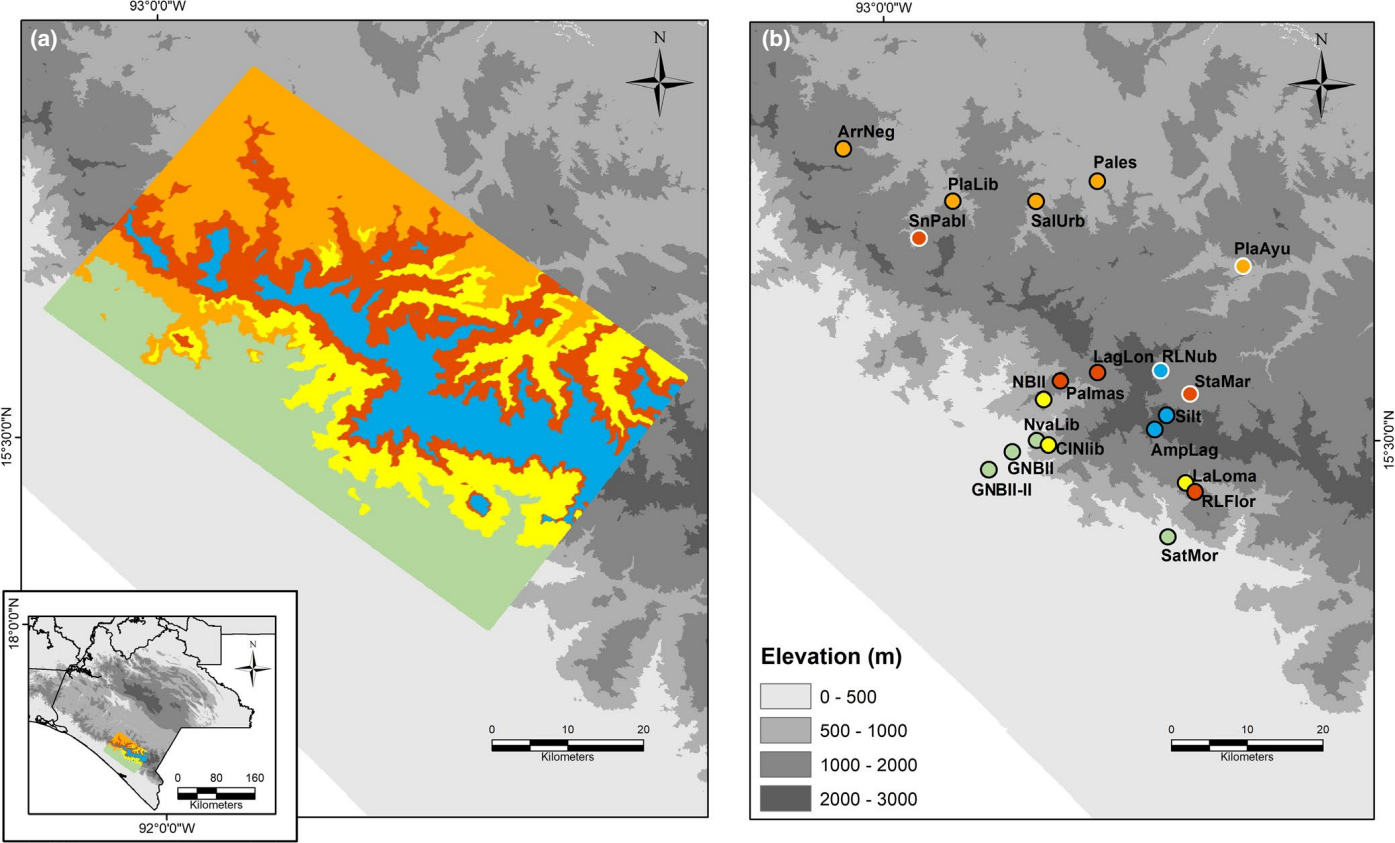
(a) Bioclimatic domains (BioDom). Green color corresponds to lowland areas and high annual temperature and precipitation. High precipitation in the warmest quarter and intermediate elevations (yellow). Intermediate elevation and temperature seasonality (orange). Higher elevations (red), and high precipitation in driest season and high elevations (blue). (b) Sampling sites of direct‐developing frog *Craugastor loki* in southern Mexico in each BioDom. Circles with white outline are sampled sites where frogs were not found

The Sierra Madre de Chiapas, Mexico, is a physiographical region with elevation gradients on both slopes: Pacific versant and the interior slopes leading to the Central Depression of Chiapas, ranging from lowland humid forest to montane cloud forest. We characterized the area using bioclimatic domains as a proxy for environmental heterogeneity (Londoño‐Murcia et al., 2010; Téllez‐Valdés et al., 2010). We outlined rectangular polygons ca 40 × 70 km at the Sierra Madre de Chiapas that covered different environments and elevation gradients (Figure 1a). We used the 19 bioclimatic variables (Table S1) from Cuervo‐Robayo et al. (2014) to determine five bioclimatic domains (Figure 1a), which are defined as *n‐*dimensional space groupings, where biological processes occur (Londoño‐Murcia et al., 2010). These bioclimatic domains are the combination of the 19 bioclimatic variables and differ from each other in temperature, precipitation, and elevation (Table S1, Figures S1 and S2). We used a resolution of 30 × 30 m for bioclimatic variables (Cuervo‐Robayo et al., 2014). Bioclimatic domains at higher elevations have lower temperatures and lower annual precipitation than bioclimatic domains at lower elevations, which have higher temperatures and precipitation. Nonetheless, when precipitation of the driest month is low, bioclimatic domains at higher elevations are wetter compared with lower elevation ones (Table S1). In each bioclimatic domain, we identified three sampling sites and visited nineteen localities along the elevation gradient from 120 m to 2,250 m a. s. l. However, we did not find frogs in all localities (Figure 1b). In total, we studied fifteen populations of *Craugastor loki* from the Sierra Madre de Chiapas during the rainy season (June to November), when frogs are most active (Figure 1b), in 2017 and 2018. For each population, we sampled frogs at night (20:00 hr–01:00 hr) using visual encounter surveys and locating frogs by their advertisement calls (Heyer et al., 1994). Occasionally, we sampled individuals during daytime hours. Once we found a frog, we approached the frog carefully, to avoid them moving or escaping and immediately measured body temperature (*T_b_*) on the dorsal side using the thermocouple of the Fluke model 51‐2 contact thermometer during 3–5 s. We also measured the substrate temperature and air temperature (20 cm above the substrate) where the frog was encountered, if the frogs moved, we only recorded substrate and air temperature. We also measured the local environmental temperature and humidity using data loggers (HOBO Onset) programmed to collect data every 30 min to estimate habitat temperature (see warming tolerance section below).

We brought captured frogs to our field laboratory for experimental trials. The frogs were kept for 3 days in plastic containers with the original substrate at laboratory temperature (~23°C) and environmental humidity. During the following 3 days, we recorded preferred temperature (*T_pref_*) on day 1, critical thermal minimum (*CT_min_*) on day 2, and critical thermal maximum (*CT_max_*) on day 3. Given data collection was stressful for the animals, we divided it into 3 days to give individuals respite between trials. Due to the short amount of time we kept frogs in the laboratory, not allowing them to acclimate to a different temperature regime, we assumed that measurements reflect responses to field conditions as a response to plasticity or local adaptation (von May et al., 2017). After all experiments, we took a toe sample from each organism and in some cases for voucher specimens we extracted the liver for genomic studies.

### 2.2 Operative temperatures

The operative temperature (*T_e_*) is defined as the steady‐state temperature of the organism; it is a proxy of the environmental heterogeneity as perceived by the individual (Angilletta, 2009). To estimate this parameter, we used physical models (Navas & Araujo, 2000) similar to typical *Craugastor* individuals using agar models of the same body size and similar body shape from latex molds made from rubber models using *C. loki* museum specimens. Models were made with granulated agar (Thermo Fisher Scientific), using 2.2 g into 100 ml of distilled water in which a data logger (Ibutton Thermochron, 4K) was placed. At each locality, we used 10 operative models, which were programed to record the temperature every 10 min for 24 hr. The ten frog models were placed in the microhabitats where *Craugastor* frogs were found including on top and under leaf litter, small cavities on trunks, and under fallen trunks.

### 2.3 Preferred temperature

The preferred temperature is based on the temperature chosen by the organism in the absence of biotic and abiotic limitations (Angilletta, 2009). It can also be considered as an estimate of optimal body temperature that maximizes life‐time reproductive success under unconstrained conditions (Gvoždík, 2018). We built two thermal gradients (1.0 m × 0.8 m and 0.7 m high) using wood boards. We used litter collected from each sampling locality as substrate. One end of the wood board was cooled and maintained at 8°C with ice packs, and the other was heated to 38°C with an electric hot plate. We ensured that substrate humidity was moderate along the entire gradient throughout the entire duration of the trials. We placed five individuals in each thermal gradient at a random location 1 hr before the start of the trials. We performed the trials at night, starting at 21:00 hr as it is the frogs' activity period. Each hour we recorded the preferred temperature (*T_sel_*) of ten individuals in both gradients, for a total of five measures for each individual (five replicates), this process was carried out for each individual in the dark and using a red light and measuring temperature with a Fluke model 51‐2 contact thermometer during 3–5 s. Each individual was weighed and measured (body and tibia length) prior to each trial.

### 2.4 Thermal accuracy index and quality index

We calculated the thermal accuracy (*d_b_*) and thermal quality index (*d_e_*) of the environment (Hertz et al., 1993) for each population. We used these two parameters to characterize the thermal landscape that the frogs are occupying, but not to evaluate the effectiveness of thermoregulation. Amphibians depend on moisture in addition to temperature does not take into account these two parameters, but it is a proxy of how frogs are suited to the thermal landscape. To estimate the *d_b_* and *d_e_*, we considered the data filtered by frog activity hours, based on our observations during fieldwork. We considered that activity starts from dusk to dawn, that is, from 18:00 hr to 5:00 hr. Thermal accuracy (*d_b_*) was calculated as the mean of the absolute value of the difference between individual body temperature (*T_b_*) and *T_sel_* (*d_b_* = *T_b_* − *T_sel_*), while the thermal quality index (*d_e_*) is the mean of the absolute value of the difference between the operative temperature (*T_e_*) and *T_sel_* (*d_e_* = *T_e_* − *T_sel_*). High *d_b_* and *d_e_* values indicate that thermal accuracy and the thermal quality of the environment are low, while values near zero indicate high thermal accuracy and high thermal quality of environment, suggesting that under such thermal conditions the organism presents increased physiological performance (Hertz et al., 1993).

### 2.5 Thermal tolerance: *CT_min_* and *CT_max_*

Thermal tolerance range or thermal breath is the physiological range at which the organism is active and is limited by the critical thermal minimum (*CT_min_*) and the critical thermal maximum (*CT_max_*). We estimated the *CT_min_* using two containers. In the first one, we deposited a damp paper before the frog was placed. The container was placed within a larger container with ice to lower the temperature at a rate of ~1.5°C/min. After some minutes, the organism changes its behavior; first, frogs move and jump and then stay very still, at which point we measured whether there was a loss of righting response. The loss of righting response is often used in thermal physiology in ectotherms and is considered relevant in terms of thermal selection, where frogs could be vulnerable to predation (Catenazzi et al., 2014; von May et al., 2017, 2019; Navas et al., 2007). To do this, we placed the frog in a belly‐up position, and if the frog could not recover its natural position, after 5 s, we measured the temperature on the ventral side. For *CT_max_*, we used an electric hot plate to heat the container, with a humid substrate. Generally, the behavior displayed when the organism starts to warm up and to reach *CT_max_* includes fast movements and jumping and then the organism stays still in one position. Previous to each experiment, for both *CT_min_* and *CT_max_* the organisms were kept at ~23°C. With the values of the *CT_max_* and *CT_min_*, we calculated the Thermal Tolerance Range (TTR = *CT_max_* − *CT_min_*).

### 2.6 Warming and cooling tolerance

One approach to measure vulnerability to climate change is to measure the warming tolerance of organisms (Deutsch et al., 2008), which is an estimate of the difference between ambient temperatures and the organism's thermal maximum (Tuff et al., 2016). Warming tolerance is similar to the Thermal Safety Margin (TSM) from Rohr et al. (2018), but it is different according to Deutsch et al. (2008), who consider it as the difference between an organism's thermal optimum (*T_opt_*) and its current climate temperature *T_hab_* (TSM = *T_opt_* − *T_hab_*). Environmental temperature from the local sites was obtained from the data logger (HOBO's) in each locality. The data loggers were set up to record temperature every 30 min daily for a period of approximately 1 year and in some cases 2 years. In some localities, recording started in 2017 and in other localities in 2018. In two localities (GNBII‐II and Palmas), it was not possible to obtain the recording, due to loss or damage of the datalogger. For these localities, we used the information from the datalogger closest to the locality (GNBII and LagLon, respectively). We estimated the average of the maximum and minimum daily temperatures during the recording period to estimate the temperature of the habitat at each locality (*T_hab_*) (Catenazzi et al., 2014). We estimated the warming tolerance (WT) as WT = *CT_max_* − *T_hab_max_* and the cooling tolerance, using the daily minimum temperature to calculate the *T_hab_min_* temperature.

### 2.7 Data analyses

We carried out linear models, to evaluate the effects of the elevation and bioclimatic domain on *T_b_*, substrate and air temperature, *CT_min_*, *CT_max_*, *TTR*, *WT*, and cooling tolerance, as well as the relationship between these variables and our microclimatic temperature measurements (*T_max_hab_*, *T_min_hab_*, *T_mean_hab_*). In consequence, we added to the linear models the body size (snout‐vent length) and mass. For *T_sel_*, we used the gls function implemented in the nlme package (Pinheiro et al., 2020), to fit a linear model with heterogeneous variances using the arguments: weights and varIdent (Pinheiro & Bates, 2000), organized hierarchically by individuals and then by population from low to high elevation. For *d_b_* and *d_e_* indexes, we calculated the confidence intervals with bootstrap (10,000 replicates). All statistical analyses were performed using the software R v 3.6.1 (R Core Team, 2020).

## 3 RESULTS

We observed 337 frogs (272 at night and 62 during the day). We measured *T_b_* in the field for 303 frogs and obtained 337 measures for substrate and air temperature (i.e., for 34 frogs which moved prior to us being able to obtain body field temperatures, we only recorded the substrate and air temperature). Field *T_b_* estimates for each locality varied from seven to 29 organisms (Table 1).

**TABLE 1:** Mean ± *SE* for the body temperature (*T_b_*), operative temperature (*T_e_*), and preferred temperature range (*T_sel_*) of individuals from different populations by year, elevation, and bioclimatic domain (colors correspond to those in Figure 1)

Year	Bioclimatic domain	Elevation	Pop *N*	*T_b_*	*T_e_*	*d_b_* (95% CI)	*d_e_* (95% CI)	Mean of *T_sel_ n*	1st & 3rd quartile of *T_sel_*
2017	– Green	124.3	GNBII‐II 10	26.33 ± 0.51		4.88 (4.65–5.25)		20.57 ± 1.60	19.60–21.45
		242.24	GNBII 17	23.72 ± 0.79	23.17 ± 1.4	2.34 (1.82–2.52)	1.82 (1.2–1.6)	21.03 ± 1.06 (10)	20.3–21.38
2017		300	SatMor 24	23.81 ± 0.34	23.14 ± 0.37	0.88 (0.77–0.97)	0.27	21.77 ± 1.87 (10)	20.88–22.93
2017		768.90	NvaLib 29	21.98 ± 0.74	22.38 ± 0.71	0.09	0.52 0.0–0.5)	21.46 ± 2.17 (11)	20.30–22.50
2018		783.61	NvaLib 20	22.33 ± 1.18	22.30 ± 0.92	0.84 (0.04–1.43)	0.81 (0.4–0.9)	23.97 ± 1.66 (10)	22.98–25.0
2018	– Orange	731.08	Pales 12	20.67 ± 0.54	20.73 ± 0.70	1.13 (0.70–1.45)	0.73 (0.3–0.3)	22.70 ± 1.38 (10)	21.80–23.75
2018		836.64	SalUrb 11	21.63 ± 0.77	20.94 ± 0.95	0.57 (0.0–0.6)	0.26	20.20 ± 1.30 (10)	19.20–21.20
2017		912.27	PlanLib 11	20.07 ± 0.73	20.73 ± 0.91	0.00	0.01	20.78 ± 1.99 (12)	19.20–22.50
2017		1,072.88	ArrNeg 8	20.07 ± 0.60	19.74 ± 9.84	0.92 (0.40–1.70)	1.29 (1.0–1.5)	21.68 ± 1.47 (7)	21.0–22.70
2017	– Yellow	1,143.87	CINLib 10	20.61 ± 0.90	19.43 ± 0.98	2.79 (2.55–3.30)	3.97 (3.9–4.4)	23.86 ± 1.13 (12)	23.40–24.70
2018		1,206.18	CINLib 11	18.41 ± 0.35	17.56 ± 0.63	4.39 (4.2–4.5)	5.24	23.52 ± 1.46 (11)	22.80–24.20
2017		1,131.82	NBI 26	20.55 ± 1.23	19.49 ± 0.75	1.07 (0.3–1.7)	0.26	18.94 ± 1.20 (10)	18.35–19.60
2018		1,128.50	NBI 20	20.56 ± 1.98	19.13 ± 1.58	0.69 (0.0–1.7)	0.78 (0.7–0.7)	20.29 ± 1.81 (12)	19.20–21.42
2017		1,303.67	LaLoma 15	18.85 ± 0.78	19.25 ± 0.70	3.45 (2.9–3.8)	3.05	22.67 ± 1.23 (10)	22.3–23.30
2018		1,298.0	LaLoma 3	17.23 ± 0.15	17.57 ± 1.03	1.67 (1.5–1.8)	1.37	19.90 ± 1.74 (6)	18.90–20.20
2018	– Red	1,380.77	Palmas 5	18.68 ± 0.52	17.91 ± 0.88	1.87 (1.25–2.65)	2.64 (2.5–3.0)	21.21 ± 1.30 (11)	20.55–22.20
2018		1,471.88	LagLon 11	18.49 ± 1.17	18.21 ± 1.26	2.21 (0.8–2.9)	2.53 (2.55–3.0)	21.58 ± 1.50 (12)	20.70–22.32
2017		1,510.0	RLFlor 22	17.68 ± 1.40	17.47 ± 0.49	4.25 (3.53–5.03)	4.46	22.93 ± 2.02 (10)	21.93–24.6
2018		1,406.80	RLFlor 5	19.54 ± 0.98	17.90 ± 0.91				
2017	– Blue	2,111.17	AmpLag 22	14.76 ± 0.42	14.25 ± 0.66	9.24 (9.10–9.45)	9.75	24.66 ± 2.30 (12)	24.0–25.80
2018		2,069.33	AmpLag 3	14.90 ± 0.79	13.53 ± 1.07	5.20 (4.3–5.8)	6.57	20.80 ± 1.28 (11)	20.10–21.60

Note

*N* is the number of the frogs sampled for the *T_b_* and *d_b_*, while *n* is the number of sampled frogs for each population for *T_sel_*. Thermal precision index (*d_b_*) and thermal quality index (*d_e_*) for each population.

Populations where the *d_b_* index is close to zero indicate that the body temperature is close to the microenvironmental temperature. Likewise, *d_e_* index near to zero indicates environmental thermal quality is very close to body temperature (*T_b_*). Conversely, if *d_b_* and *d_e_* are different from zero, body temperature is far from microenvironmental temperature and thermal quality is not favorable to frogs. *d_b_*, *d_e_*, and *T_sel_* are described in Materials and Methods.

### 3.1 Body, substrate, and air temperature

We found a linear decrease in body temperature with increasing elevation (*F*_20,177_ = 144.1, *R* ^2^ = 0.935, slope = −0.006 ± 0.03, *p* < .0005, Table S2, Figure S3a). Substrate and air temperature also exhibited the same pattern with increasing elevation (*F*_19,266_ = 125, *R* ^2^ = 0.892, slope = −0.0019 ± 0.0018, *p* < .0005; *F*_19,266_ = 135.8, *R* ^2^ = 0.899, slope = −0.002 ± 0.0017, *p* < .0005, respectively; Table S2, Figure S3b,c). Both substrate and air temperatures are highly correlated with body temperature of the sampled frogs (*r* = .970, *n* = 256, *p* < .005; *r* = 0.975, *n* = 256, *p* < .005, respectively; Figure S5a,b).

### 3.2 Operative temperature and preferred temperature

**FIGURE 2:**
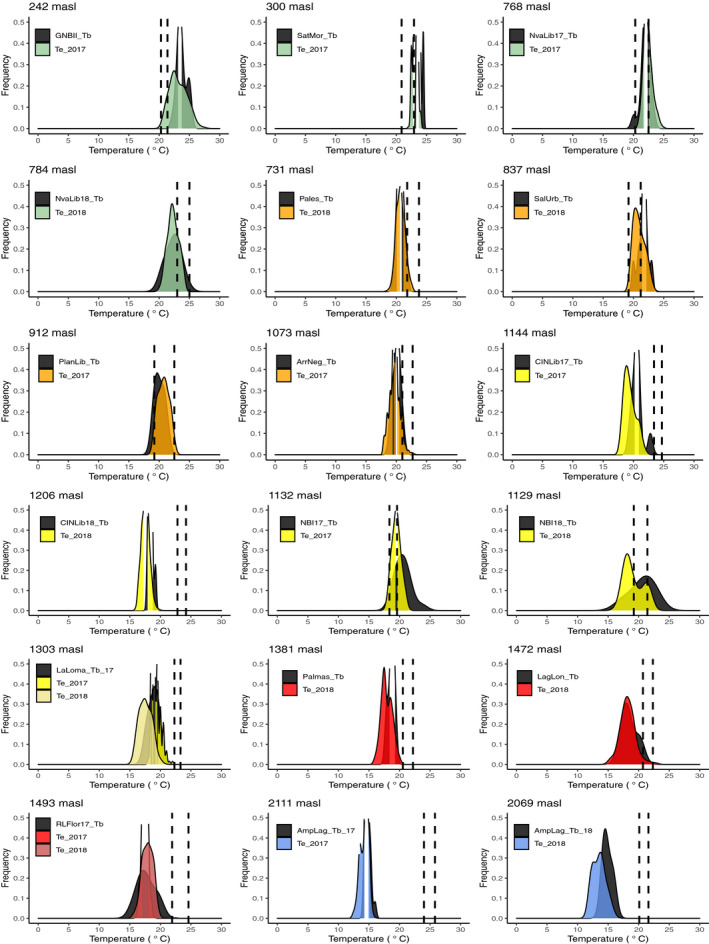
Thermal physiological traits: Body temperature (*T_b_* in black color), selected temperature (*T_sel_* dashed lines), and operative temperature (*T_e_*) in colors according to bioclimatic domains (Figure 1). Dashed lines represent the interval between 1st and 3rd quartile of *T_sel_*, *x*‐axis shows the records of *T_e_* in degrees Celsius, and *y*‐axis shows the frequency of the temperature recorded

**FIGURE 3:**
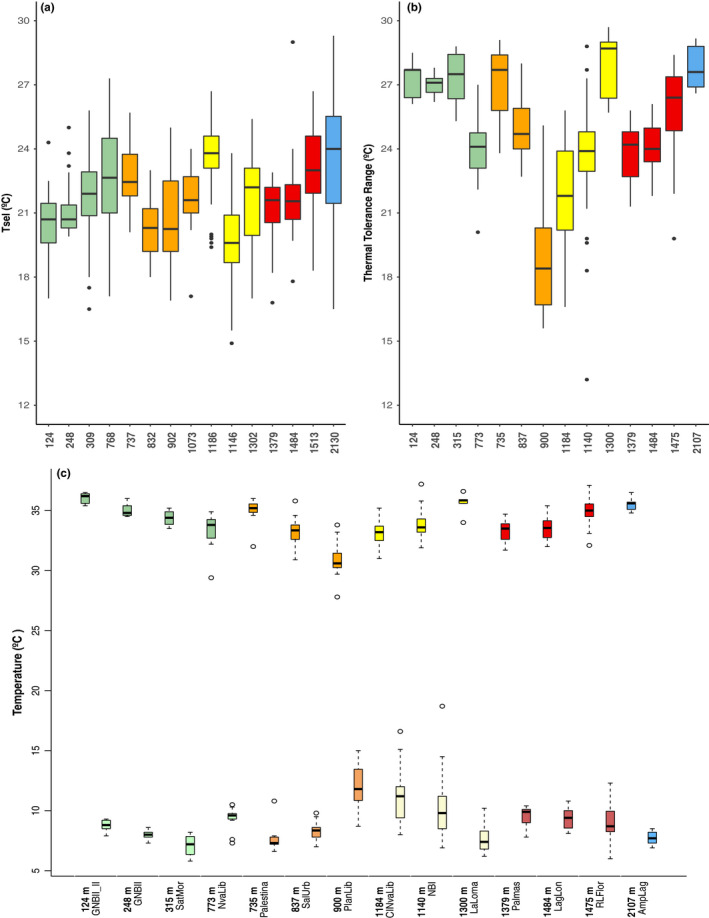
(a) Preferred temperature (*T_sel_*) and (b) Thermal tolerance range (TTR) of populations sampled along the elevational gradient and bioclimatic domain (colors). The *x*‐axis represents the elevation in meters above sea level. (c) Relationship between thermal tolerance limits in different elevations and bioclimatic domains. (*CT_max_*: top) and (*CT_min_*: bottom). The *x*‐axis represents the elevation in meters and the name of population sampled

The operative temperature (*T_e_*) was similar to the body temperature (*T_b_*) in all populations along the elevation gradient (Table 1, Figure 2). Thus, *T_e_* shows a similar pattern to body temperature (*T_b_*); that is, *T_e_* decreases as the elevation increases (Figure 2). The preferred temperature (*T_sel_*) for each population varied with elevation (*F*_13,930_ = 25.51, *p* < .0001; Table S3, Figure 3a) and differed between the bioclimatic domains (*F*_4,930_ = 9.29, *p* < .0001; Table S3, Figure 3a). We also found that body size (snout‐length SVL) is positively correlated with *T_sel_*. Smaller frogs tend to select a relative lower temperature compared with larger frogs (*F*_1,930_ = 7.97, *p* = .0049, Table S3, Figure S6a). When we pooled populations, the mean of *T_sel_* is 21.83 ± 2.18°C, and the minimum and maximum means of *T_sel_* are 19.60 ± 1.70°C and 23.70 ± 1.30°C, respectively. The *T_sel_* recorded in the lowlands and at intermediate elevations (bioclimatic domains: green, orange, and yellow) is within the range of the operative temperature (*T_e_*) and body temperature (*T_b_*) recorded. However, in populations above 1,100 m, *T_sel_* is outside of the range recorded for *T_e_* and *T_b_* (bioclimatic domains: red and blue, Figures 2 and 3a).

### 3.3 Thermal accuracy and thermal quality indexes

The thermal accuracy index (*d_b_*) and thermal quality of the environment index (*d_e_*) showed lower values in all populations from intermediate elevations (760–1,140 m, corresponding to the orange bioclimatic domain), meaning that in this bioclimatic domain the frogs are exposed to their preferred temperature (*T_sel_*). Indeed, in one population (PlanLib) within the orange bioclimatic domain (Table 1), the estimates of both indexes were zero, indicating that in this site the frogs occupy more suitable temperatures. On the other hand, the populations in higher elevations (blue bioclimatic domain) show high values of *d_b_* and *d_e_*, indicating that these populations are exposed to environmental temperatures that differ from the preferred temperature. Also, some populations at intermediate elevations (yellow bioclimatic domain) present high values of *d_b_* and *d_e_* (Table 1).

### 3.4 Critical thermal limits

The critical thermal maximum (*CT_max_*) differs among populations at different elevations, decreasing with increasing elevation (*F*_19,137_ = 10.86, *R* ^2^ = 0.545, slope = −0.0118 ± 0.0042, *p* < .0056; Table S4, Figure 3c), and also differs between bioclimatic domains (*F*_4,137_ = 2.6, *p* < .038) (Figure 3c). Body size (snout‐vent length SVL) was not correlated with *CT_max_* (*F*_1,137_ = 1.64, *p* = .20). We also found differences in *CT_min_*, among populations (*F*_19,139_ = 7.29, *R* ^2^ = 0.43, slope = 0.0044 ± 0.0062, *p* < .0001; Table S4, Figure 3c), decreasing with increasing elevation, but no differences among bioclimatic domains (*F*_1,137_ = 1.64, *p* = .20). Body size (SVL) is negatively correlated with *CT_min_*, with small frogs having higher *CT_min_* (*F*_1,139_ = 11.79, *p* < .001; Table S4, Figure S7). The thermal tolerance range (*TTR*) slight increases with elevation (*F*_19,136_ = 11.98*, R* ^2^ = 0.573, slope = −0.0162 ± 0.0081, *p* < .049; Table S4, Figure 3b), and body size has an effect on thermal breath (*F*_1,136_ = 10.62*, p* < .0014), but not bioclimatic domains (*F*_4,136_ = 0.38*, p* = .821; Figure 3b).

**FIGURE 4:**
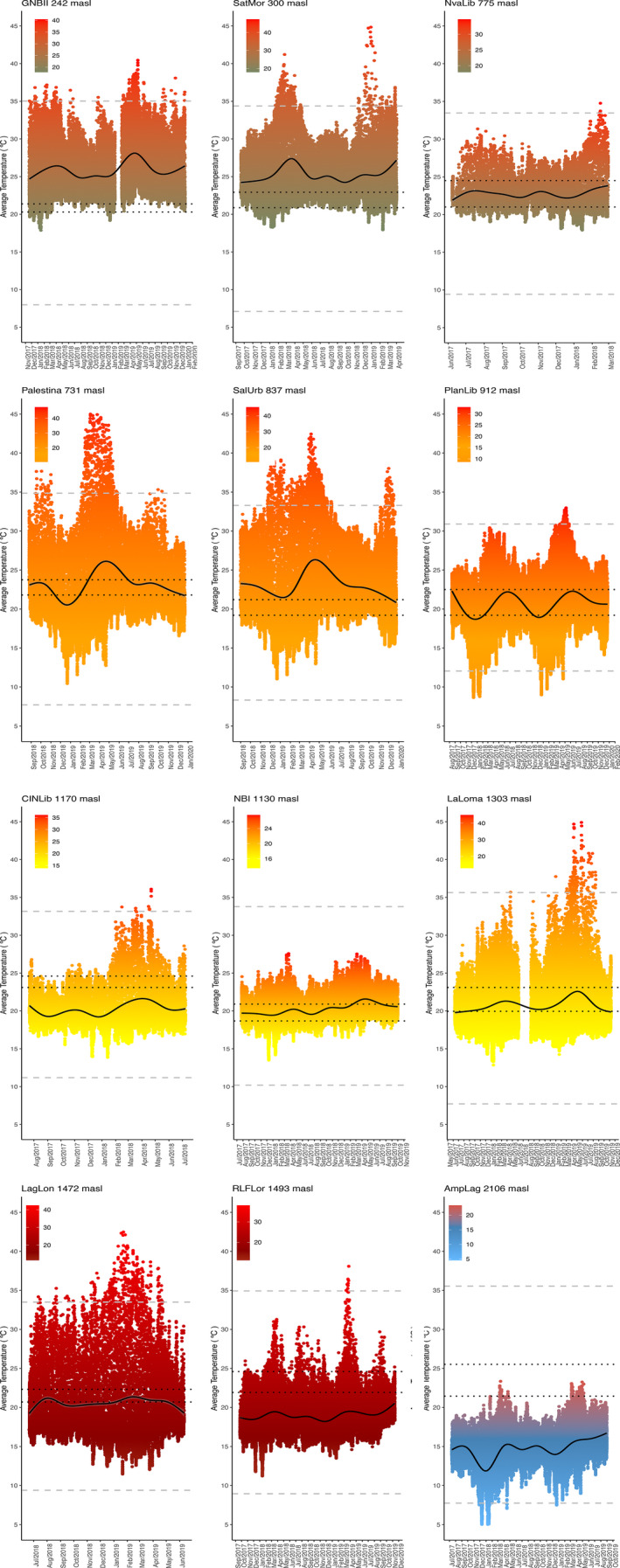
Microclimate temperatures (mean daily) and thermal limits of sampling localities between June 2017 and December 2019 at the five bioclimatic domains. Gray dashed lines represent the upper and lower thermal limits (*CT_max_* and *CT_min_*), respectively. Black dotted lines are the intervals of the *T_sel_*, and the bold line represents mean temperature

With respect to the local environmental data gathered by the data loggers, we found a 10°C difference between the extreme low and high populations (mean = 25°C ± 3.45 and 14.70°C ± 2.35, respectively; Table S5). On the other hand, we observed a wide range of recorded temperatures for some sites (Figure 4), where the daily temperature can exceed the range of critical thermal limits (Figure 4). This happens in more than 60% of the sampled localities (Figure 4).

There was not a significant relationship between the physiological thermal limits (*CT_max_*) with respect to local environmental temperature (*T_max_hab_*) (*F*_1,137_ = 0.22, *p* = .63), but we found differences between in elevation by bioclimatic domain (*F*_19,137_ = 10.86, *R* ^2^ = 0.5415, slope = −0.012 ± 0.004, *p* < .05, Table S4, Figure S8a). Furthermore, we did not find a relationship between *CT_min_* with *T_min_hab_* (*F*_1,139_ = 0.74, *p* = .39, Table S4, Figure S8b). When we plotted the *CT_max_*, *CT_min_*, and the elevation with bioclimatic domain, we found decreasing *CT_max_* as *CT_min_* increases (*F*_20,135_ = 10.73, *R* ^2^ = 0.556, slope = −0.123 ± 0.057, *p* < .0001, Table S4, Figure S9). This could be a consequence of the intrapopulation variability in both thermal limits, but it could also be driven by body size (see discussion).

### 3.5 Warming and cooling tolerance

**FIGURE 5:**
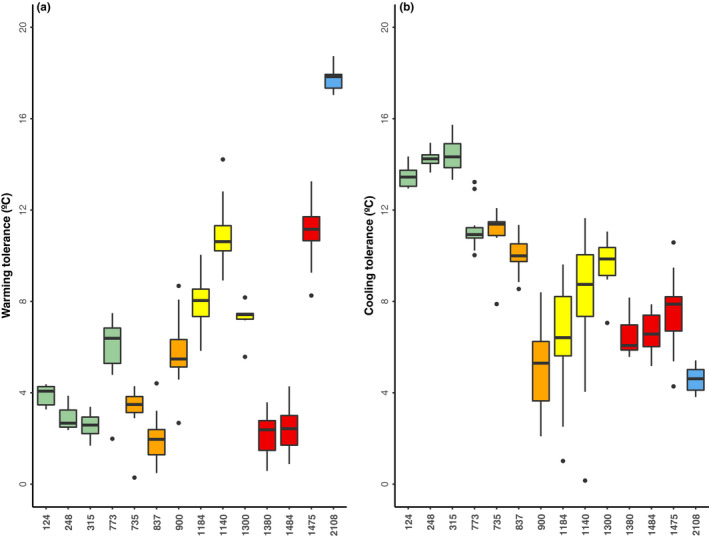
Warming and cooling tolerance of *Craugastor loki* along the elevational gradient. Colors correspond to each bioclimatic domain (Figure 1)

Populations from low elevations (green bioclimatic domain) have lower warming tolerance (*F*_19,137_ = 143.1, *R* ^2^ = 0.944, slope = −0.0114 ± 0.0041, *p* < .0001, Table S4, Figure 5a) than those from other bioclimatic domains. Conversely, populations from high elevations have lower cooling tolerance, in particular the populations at elevations ~1,370 m–1,500 m (*F*_19,139_ = 28.66, *R* ^2^ = 0.769, slope = −0.0044 ± 0.0061, *p* < .0001, Table S4, Figure 5b), which are within the red bioclimatic domain.

## 4 DISCUSSION

We have shown that thermal sensitivities of the direct‐developing *Craugastor loki* are influenced by the particular environmental conditions where the populations are found, as defined by elevation and the bioclimatic domains. Our results show there is greater plasticity in critical thermal maxima (*CT_max_*), compared with critical thermal minimum (*CT_min_*), as *CT_max_* shows greater variability in response to changes in elevation or bioclimatic domain. The *CT_max_* is higher in the lowlands and tends to decrease as elevation increases. Also, it is noteworthy that the *T_sel_* exhibits a narrow range along the elevation gradient and even tends to increase with elevation within each bioclimatic domain.

### 4.1 Body temperature

Body temperature (*T_b_*) decreases significantly with increased elevation, which is a pattern found in most ectotherms that do not exhibit thermoregulation behavior (Angilletta, 2009). Tropical amphibians, especially terrestrial frogs, are highly dependent on the substrate and local environment for their body temperature (Navas, 1996). This pattern has also been found in *C. loki*, where *T_b_* is highly correlated with substrate and air temperature, confirming that *C. loki is* a thermoconforming species. The *T_b_* influence on individual performance (Navas et al., 2008) impacts activities such as foraging, courtship, and locomotion. As a consequence, *T_b_* is important as a trait for resilience to climate variability. On the other hand, if *T_b_* is associated with environmental variables (Navas et al., 2013), it implies differences in the effects of microclimate on individual performance and traits (Clusella‐Trullas & Chown, 2014) such as development, growth and performance, all of which impact fitness (Kingsolver & Huey, 2008). Also, *T_b_* is linked to other parameters such as preferred temperature (*T_sel_*) and critical thermal limits, which are considered as traits integrated in the species thermal niche (Gvoždík, 2018).

In our study, the relationship between *T_b_*, and *T_sel_* varies across the elevation gradient and bioclimatic domain. In lowland populations, *T_sel_* is relatively lower than the range of *T_b_* (GNBII and SatMor, Figure 2). At intermediate elevations (orange environmental domain, Figure 2), *T_sel_* is within the range of *T_b_*, and in one population (PlanLib) *T_sel_* matches *T_b_* exactly. As elevation increases, part of the narrow range of *T_b_* falls within the range of *T_sel_*. Since these thermal parameters partly define the thermal niche, population growth and development are expected to be affected (Gvoždík, 2018). Our field observations of highland populations indicate that the reproductive season starts later compared with the remaining populations (at lower altitudes) and has a shorter duration, which indirectly affects population growth. Disentangling how the thermal niche could impose costs directly to population growth and development is an important question to be addressed in future work, particularly with respect to how these populations can face forthcoming climate change challenges.

### 4.2 Preferred temperature and thermal indices

The preferred temperature (*T_sel_*) in *Craugastor loki* shows a narrow range across elevations, but it tends to slightly increase as the elevation increases within each bioclimatic landscape (Table 1). This increase is important given that some studies show that *T_sel_* is close to *T_b_* or even near the upper thermal limit (Angilletta, 2009). Our findings show that *T_sel_* tends to slightly increase across the elevational gradient; however, in the highest populations *T_sel_* is closer to the *T_sel_* recorded in lowland sites. These results are similar to studies of nocturnal geckos, some of which are thermoconforming species (Pianka & Vitt, 2003) and tend to have a constant *T_sel_*. In our study, the *T_sel_* mean for each sampled population was between 18.94 ± 1.20 and 24.66 ± 2.30, with the latter value recorded in the high‐elevation population (Table 1, Figure 3a). *T_sel_* is generally considered a conserved trait among species (Anderson et al., 2018). Here at the population level, differences in *T_sel_* show a narrow range along all elevation localities. These observed variations could be the results of interpopulation variation in *T_sel_*. However, in highland populations where the mean average temperature is less than 20 degrees Celsius, the *T_sel_* of frogs was ~5 degrees higher than the air temperature, which could be related to a pattern known as counter gradient variation. This pattern is observed when individuals from colder environments select higher temperatures in comparison with individuals from hot environments (Freidenburg & Skelly, 2004). Also, counter gradient has been related to the organisms having higher *CT_max_* (Llewelyn et al., 2017). Further work is necessary to evaluate whether this hypothesis can be applied to frogs and *Craugastor* in particular. In addition, highland populations prefer higher temperatures than they experience, suggesting that this imposes an important challenge for individuals. These data suggest impacts on their “performance” and on fitness. During sampling in the highland population (AmpLag), we saw some frogs active during the day, not calling, but moving among the sunny patches. These frogs were possibly looking for warmth and staying in suitable microhabitats, which suggests that at high elevations frogs have to deal with thermal niche by exhibiting behaviors not shown in the lowlands.

On the other hand, it is important to point out that our preliminary studies on landscape genomics for these populations suggest that one highland population (AmpLag; blue environmental domain) might correspond to a divergent evolutionary lineage. Therefore, the maximum elevation range of *C. loki* might only extend to ~1,500 m a. s. l. and populations above that elevation could correspond to another species (Fig. S10). In the highlands of the Sierra Madre de Chiapas, at least four species of *Craugastor* that are very similar in coloration, size, and habits are believed to occur. Therefore, the sampled highland populations (blue environmental domain) might be similar to *C. loki* from higher elevations (red domain) in terms of thermal landscape. Effectively, the populations in each elevational extreme and bioclimatic domain confer the same thermal pressures on these physiological traits.

The results from the thermal accuracy (*d_b_*) and the thermal quality of the environment (*d_e_*) show that the populations from the orange environmental bioclimatic domain (characterized by the temperature seasonality at intermediate elevations) have high thermal accuracy and thermal quality. These conditions seem optimal for the frogs, given their preferred temperature is available in that bioclimatic landscape. It is noteworthy that one population (PlanLib) shows estimates close to zero for both indices, indicating remarkable thermal quality. Those frogs do not have to deal with extreme temperatures as they do in the lower or higher elevations, experiencing close to an ideal thermal niche. These thermal parameters used mainly in lizards studies have proven useful in understanding the thermal landscape in frogs as well. Our data at the population level permit the splitting of thermal niche and how it can vary with spatial and temporal scale (Gilbert & Miles, 2019). While we did not take into account humidity, our sampling was performed in rainy season where the humidity is mainly similar and oscillated between 90% to 100% humidity (Table S6). As a result, understanding the thermal niche in these frogs is a step toward understanding the causes that produce phenotypic variation in thermal traits in buffering high temperatures and coping with rising temperatures.

### 4.3 Critical thermal limits

The critical thermal limits measured in our study showed thermal acclimatization on the upper thermal limit associated mainly with altitude and the environment, though patterns were less clear in lower thermal limits. That is, within each of the sampled bioclimatic domains as elevation increases, the upper thermal limit decreases, especially in populations from 200 m to 1,000 m a. s. l. In most interspecific studies of ecthotherms, species show that upper thermal limit is a conserved trait among species or within a particular species (Muñoz et al., 2014). Likewise, in frogs of the genus *Pristimantis* along an elevation gradient in the Andes, *CT_min_* is more variable than *CT_max_* by elevation. This finding is explained by microclimatic thermal variation, mainly in minimum temperatures, suggesting that this group of frogs has increased cold tolerance to buffer against lower temperatures (Pintanel et al., 2019). However, another study focused on terrestrial breeding frogs distributed along a tropical elevation gradient has shown that *CT_max_* and *CT_min_* exhibit substantial variation across closely related species (von May et al., 2017), where *CT_max_* and *CT_min_* decrease with increasing elevation. Other studies found interspecific variation in critical thermal limits related to body size, where *CT_max_* tends to increase with increasing body size (González‐del‐Pliego et al., 2020; von May et al., 2019), and *CT_min_* tends to decrease with increasing body size (von May et al., 2019). In our study, we only found that *CT_min_* tends to decrease as body size increases. This contrasts with interspecific studies in terrestrial breeding frogs, which suggested that larger body size is common at high‐elevation habitats (Gonzalez‐Voyer et al., 2011; Hedges, 1999; Santa‐Cruz et al., 2019). Here, it is clear that lowland populations of *C. loki* tend to have a larger body size (Figure S4). Also, we found a correlation between *CT_max_* and *CT_min_* (Figure S9), which could be the consequence of body size. That is, intrapopulation variability in both thermal limits could be driving by body size, and results in larger individuals tend to have higher *CT_max_* and lower *CT_min_* (larger individuals better tolerate heat and cold, because they are less susceptible to relatively rapid temperature change). While smaller individuals tend to have lower *CT_max_* and higher *CT_min_*, because they are more susceptible to relatively rapid temperature change.

The role of critical thermal limits in ectotherms has an ecological importance (von May et al., 2019). This was a consequence of the organisms experiencing temperatures closer or exceeding *CT_max_*, which could result in death. But in the case of *CT_min_*, the organism does not necessarily face death as becoming more inactive. However, even when temperatures are low *C. loki* is active nocturnally. During the day spend, their time apparently inactive and are exposed to elevated air temperatures in leaf litter, where the *T_hab‐max_* can be very high in every environmental domain independent of elevation. Microclimatic temperatures at intermediate and high elevations can reach high temperatures similar to those recorded in lowland sites and at night lowlands are usually warmer but show greater variability during the day (Ghalambor, 2006).

According to the climatic variability hypothesis (Janzen, 1967), climatic variation and physiological tolerance in tropical and temperate regions are considered equivalent to conditions in elevation gradients (Ghalambor, 2006). We therefore expected lowland populations to present higher thermal limits than populations from the highlands. At an interspecific level, we expect that environmental variation could result in an adaptive change, while at the intraspecific level, variation could be due to thermal plasticity instead of an adaptive response. However, the role of plasticity could be more important. Thermal tolerance range (*TTR*) in *C. loki* exhibits a different pattern than expected across the elevation gradient. Populations at low and high elevations have narrower *TTR*, while at intermediate elevations, TTR is wider. Microclimatic temperatures could explain this pattern, as the average maximum temperature (*T_max‐hab_*) in lowlands and highlands are relatively similar. That is, *T_max‐hab_* do not show a pattern with respect to elevation and neither does the bioclimatic domain, while *T_mean_* and *T_min_hab_* both show a slight decrease with elevational increase. This trend is not shown in *T_max‐hab_*, where in lowlands (i.e., GNBII, 300 m elevation), *T_max‐hab_* can reach around 30°C, as well as in highlands (i.e., LagLon, 1,500 m elevation).

Studies with other terrestrial breeding frogs suggest that thermal limits are related to microclimate temperatures (González‐del‐Pliego et al., 2020; Pintanel et al., 2019). That is, thermal limits exhibit variation according to the type of microhabitat they occupy including open forests (González‐del‐Pliego et al., 2020; Nowakowski et al., 2017, 2018). In our study, *C. loki* is mainly found in leaf litter (more than 70% of the sampled individuals); therefore, microhabitat temperatures, especially at night, do reflect temperatures to which frogs are usually exposed. This is probably not the case at daytime when they are inactive in the leaf litter and temperatures are higher. As a consequence, further work is needed to test how habitat modifications affect thermal traits. This is particularly critical since our study area has changed drastically over the course of the study (Percino‐Daniel, pers. obs.), especially at intermediate elevations where coffee plantations and open areas are increasing.

### 4.4 Vulnerability to climate change

Our study provides insights regarding the sensitivity of these terrestrial frogs to climate change. First, our results show that lowland populations can be more vulnerable to high daily temperatures due to low warming tolerance (Figure 5a) while highland populations could benefit or be less vulnerable to temperature increases. Macroecological studies suggest that tropical organisms are more vulnerable to climate change for two reasons: (a) They experience environmental temperatures near their critical tolerance temperatures (Deutsch et al., 2008; Kingsolver, 2009); and (b) they have a narrow thermal tolerance (Janzen, 1967) and are therefore more sensitive to changes in climate. Here, at a fine scale we found that lowland frogs are more vulnerable to warming due to their limited thermal tolerance. Also, populations at high elevations are less vulnerable (bioclimatic blue domain). However, these findings can vary within each bioclimatic domain. For example, populations from elevations sampled ca ~1,300–1,400 m also exhibited a low warming tolerance (Figure 5a) and these sites experience high temperatures (Table S2). Previous research on other terrestrial breeding frogs includes species distributed from mid‐ to high elevations (Catenazzi et al., 2014), as well as species distributed from low to high elevations (González‐del‐Pliego et al., 2020; von May et al., 2019). However, the maximum daily temperature recorded at some sites in our study reached above 40°C (Table S2), which are dangerously high temperatures for thermoconformers. Indeed, IPCC reports (2019) predict the global temperature will increase to or exceed ~1.5°C, making it critical to understand how the frogs will respond to this warming increase. Hence, integrating an approach from a species' niche rather than a traits‐based approach could make for a better understanding of general patterns of tolerance (Frishkoff et al., 2015).

In conclusion, our findings indicate that the future increase in global temperatures is likely to negatively affect performance and population growth of thermoconforming species such as *C. loki*. Predictive models can be used to test distinct scenarios for distribution shifts of species that integrate microclimate variables. As in this study, local temperatures can reveal if daily temperatures approach or exceed thermal limits for a species. This approach could better characterize long‐term persistence of amphibian populations.

## CONFLICT OF INTEREST

The authors declare no conflicts of interests exist.

## AUTHOR CONTRIBUTIONS

**Ruth Percino‐Daniel:** Conceptualization (lead); data curation (equal); formal analysis (equal); investigation (equal); methodology (equal); writing‐original draft (lead); writing‐review & editing (lead). **José M. Contreras López:** Data curation (equal); methodology (equal); validation (supporting). **Oswaldo Téllez‐Valdés:** Data curation (equal); methodology (equal); writing‐review & editing (supporting). **Fausto R. Méndez de la Cruz:** Conceptualization (equal); methodology (equal); validation (equal); writing‐review & editing (equal). **Alejandro Gonzalez‐Voyer:** Conceptualization (equal); methodology (equal); validation (equal); writing‐review & editing (equal). **Daniel Piñero:** Conceptualization (equal); funding acquisition (lead); project administration (lead); resources (lead); supervision (equal); validation (equal); writing‐review & editing (equal).

### Open Research Badges

This article has been awarded Open Data, Open Materials Badges. All materials and data are publicly accessible via the Open Science Framework at https://doi.org/10.5061/dryad.dr7sqv9xp.

## Supporting information

Supplementary Material

## Data Availability

The data used in this study are available for download from Dryad at https://doi.org/10.5061/dryad.dr7sqv9xp.
