# Supplementary material for: Environmental heterogeneity shapes physiological traits in tropical direct‐developing frogs
Source: Ecol Evol. 2021 May 1;11(11):6688–702. doi: 10.1002/ece3.7521 (PMC8207348; doi:10.1002/ece3.7521)
Supplement: Supplementary file 1 — Supplementary Material [file ECE3-11-6688-s001.pdf]

Supplementary material

Environmental heterogeneity shapes physiological traits in tropical direct-developing frogs

Ruth Percino-Daniel, José M. Contreras López, Oswaldo Téllez-Valdés, Fausto R. Méndez de la Cruz, Alejandro Gonzalez-Voyer and Daniel Piñero

Tables S1 to S6

Figures S1 to S10

Table S1. Mean of bioclimatic variables used for characterized the bioclimatic domains at the study area.

	Bioclimatic domains				
	- Green	- Orange	- Yellow	- Red	- Blue
Elevation (m)	376.1	976.7	1144	1571	2054
Renge of elevation	30.1 – 915.6	482 - 1552	649.6 – 1619	1180 - 2061	1514 - 2758
Bioclimatic variables					
1. Annual Mean Temperature	25.7	22.3	21.5	19.4	17.1
2. Mean Diurnal Range	12.9	12.7	11.8	11.2	10.7
3. Isothermality	0.68	0.6	0.65	0.66	0.70
4. Temperature Seasonality	0.35	0.5	0.41	0.41	0.33
5. Max Temperature of Warmest Month	35.02	32	30.18	27.48	24.48
6. Min Temperature of Coldest Month	15.87	12.1	12.23	10.70	9.31
7. Temperature Annual Range	19.14	19.9	17.94	16.79	15.17
8. Mean Temperature of Wettest Quarter	25.95	23.0	22.04	20.02	17.51

9. Mean Temperature of Driest Quarter	24.65	20.7	20.22	18.15	16.18
10. Temperature of Warmest Quarter	26.91	24.0	22.85	20.65	18.07
11. Mean Temperature of Coldest Quarter	24.53	20.3	19.97	17.89	15.88
12. Annual Precipitation	2821.13	2123.6	2539.62	2011.07	1830.02
13. Precipitation of Wettest Month	145.09	111.4	127.40	101.68	90.10
14. Precipitation of Driest Month	1.115	1.2	2.710	2.637	3.619
15. Precipitation Seasonality	93.59	97.9	91.68	90.88	84.03
16. Precipitation of Wettest Quarter	1522.96	1200.6	1336.20	1034.79	891.26
17. Precipitation of Driest Quarter	28.84	23.8	44.71	39.68	62.22
18. Precipitation of Warmest Quarter	416.72	332.1	410.40	373.53	389.40
19. Precipitation of Coldest Quarter	36.3	32.4	54.55	47.74	74.50

Table S2. Estimated values of the fitted linear model for body temperature (T_b), air temperature (AirTemp) and substrate temperature (SubsTemp) with elevation, nested by population and bioclimatic domain (biodom). The bold number represents the significance values.

BodyTemp ~ Elevation + Pop + Weight + BioDom:Elevation

**Body Temperature (T_b)**		**Estimate**	**Std. Error**	**t value**	**Pr(>|t|)**
	Intercept	26.95	0.4328	62.260	< 2e-16 ***
	Elevation	-0.006	0.0028	-2.034	0.0434 *
	GNBII	-1.935	0.4500	-4.300	2.82e-05 ***
	SatMor	-1.601	0.600	-2.672	0.00824 *
	NvaLib	-0797	1.757	-0.454	0.65072
	Palestina	0.455	3.670	0.123	0.90230
	SalUrb	2.358	4193	0.562	0.57466
	PlanLib	1.487	4.570	0.325	0.74528
	ArrNeg	3.201	5406	0.592	0.55445
	CINvaLib	-0.969	2.862	-0.338	0.73554
	NBI	0.293	2.711	0.108	0.91402
	LaLoma	-0.250	3.140	-0.079	0. 93675
	Palmas	20.906	7.462	2.801	0.00565 **
	LagLon	22.761	7.974	2.855	0.00483 **
	RLFlor	22.870	8.210	2.786	0.00593 **
	AmpLag	-7.135	9.477	-0.753	0.45249
	Weight	0.044	0.058	0.753	0.45257
	Elevation:OrangeBiodom	-0.004	0.006	-0.640	0.52276
	Elevation:YellowBiodom	-0.0003	0.004	-0.086	0.93150
	Elevation:RedBiodom	-0.016	0.006	- 2.586	0.01050 *
	Elevation:BlueBiodom	0.0031	0.005	0.603	0.54708

SubsTemp ~ Elevation + Pop + BioDom:Elevation

**Substrate Temp**		**Estimate**	**Std. Error**	**t value**	**Pr(>|t|)**
	Intercept	27.413	0.384	71.441	< 2e-16 ***
	Elevation	-0.001875	0.002	-1.011	0.31274
	GNBII	-2.571	0.444	-5.787	2.01e-08 ***
	SatMor	-3.194	0.517	-6.177	2.44e-09 ***

NvaLib	-3.277	1.253	-2.615	0.00943 **
Palestina	0.381	4.054	0.094	0.92524
SalUrb	2.433	4.633	0.525	0.59992
PlanLib	1.660	5.047	0.329	0.74257
ArrNeg	2.727	5.931	0.460	0.64599
CINvaLib	2.836	2.61	1.083	0.2796
NBI	3.066	2.534	1.210	0.22747
LaLoma	3.198	2.914	1.097	0.27345
Palmas	20.810	4.852	4.308	2.32e-05 ***
LagLon	22.667	5.181	4.375	1.74e-05 ***
RLFlor	22.162	5.238	4.231	3.21e-05 ***
AmpLag	-0.560	11.725	-0.051	0.95926
Elevation:OrangeBiodom	-0.008	0.006	-1.310	0.19138
Elevation:YellowBiodom	-0.007	0.003	-2.450	0.01495 *
Elevation:RedBiodom	-0.019	0.004	-4.877	1.86e-06***
Elevation:BlueBiodom	-0.004	0.006	-0.642	0.52166

AirTemp ~ Elevation + Pop + BioDom:Elevation

Air Temp		Estimate	Std. Error	t value	Pr(>|t|)
	Intercept	26.695	0.359	74.341	< 2e-16 ***
	Elevation	-0.002	0.002	-1.088	0.27778
	GNBII	-2.272	0.416	-5.468	1.05e-07 ***
	SatMor	-2.686	0.484	-5.551	6.86e-08
	NvaLib	-2.893	-2.893	-2.467	0.01425 *
	Palestina	-1.903	3.794	-0.502	0.61628
	SalUrb	0.135	4.335	0.031	0.97515
	PlanLib	-1.346	4.723	-0.285	0.77581
	ArrNeg	-0.535	5.550	-0.096	0.92323
	CINvaLib	4.650	2.449	1.898	0.05872
	NBI	4.506	2.372	1.900	0.05854
	LaLoma	4.624	2.727	1.696	0.091079
	Palmas	19.816	4.540	4.365	1.82e-05 ***
	LagLon	21.069	4.848	4.346	1.98e-05 ***
	RLFlor	20.895	4.902	4.263	2.81e-05 ***
	AmpLag	0.662	10.972	0.060	0.95195
	Elevation:OrangeBiodom	-0.004	0.005	-0.737	0.46204
	Elevation:YellowBiodom	-0.008	0.003	-2.947	0.00349 **
	Elevation:RedBiodom	-0.018	0.004	- 4.877	1.86e-06 ***

Elevation:BlueBiodom	-0.004	0.005	-0.748	0.45503

Table S3. Estimated values of the fitted linear model of preferred temperature (T_{sel}) with elevation and bioclimatic domain (BioDom)

Temperature ~ Elevation + Pop + SVL + Weight + Domain:Elevation

	Value	Std error	t-value	p-value
Intercept	18.32509	2.128315	8.610140	0.0000
Elevation	-0.00263	0.015863	-0.165867	0.8683
GNBII	0.70491	2.011691	0.350409	0.7261
SatMor	1.76647	2.958486	0.597086	0.5506
NvaLib	2.43185	9.444801	0.257481	0.7969
Palestina	4.63049	3.863886	1.198403	0.2311
SalUrb	2.46680	4.221941	0.584281	0.5592
PlanLib	3.31105	4.498267	0.736073	0.4619
ArrNeg	5.01590	5.197033	0.965147	0.3347
ClNvaLib	9.24224	3.160443	2.924351	0.0035
NBI	5.28888	3.096519	1.708007	0.0880
LaLoma	7.98008	3.351273	2.381209	0.0174
Palmas	1.83505	10.101990	0.181653	0.8559
LagLon	2.26797	10.839840	0.209226	0.8343
RLFlor	3.85055	11. 052346	0.349206	0.7270
AmpLag	70.95077	9.640842	7.359396	0.0000
SVL	0.13719	0.042836	3.202741	0.0014
Body mass	-0.56566	0.237544	-2.381301	0.0174
Elevation:OrangeBiodom	-0.00119	0.016503	-0.072270	0.9424
Elevation:YellowBiodom	-0.00292	0.015979	-0.182569	0.8552
Elevation:RedBiodom	0.00156	0.017413	0.089350	0.9288
Elevation:BlueBiodom	-0.02927	0.016503	-1.773625	0.0764 .

Table S4. Estimated values of the fitted linear model of the physiological limits: Critical thermal maximum (CT_{max}), critical thermal minimum (CT_{min}), and thermal tolerance range (TTR) in relation with elevation, body size (snout-vent length SVL), and bioclimatic domain (BioDom). As well as, warming tolerance (*WT*) and cooling tolerance in relation with elevation and bioclimatic domain (BioDom) and maximum (T_{max_hab}) and minimum ($T_{min-hab}$) daily temperature.

CT_{max} ~ Elevation + Pop + SVL + BioDom:Elevation				
	Estimate	Std. Error	t value	p-value
Intercept	36.628753	0.956263	38.304	< 2e-16***
Elevation	-0.011794	0.004194	-2.812	0.00565 **
GNBII	0.513487	0.783067	0.665	0.50688
SatMor	0.717410	1.063065	0.675	0.50091

NvaLib	5.190772	2.770644	1.873	0.06313
Palestina	-5.120810	4.173799	-1.227	0.22197
SalUrb	-7.073525	4.748451	-1.490	0.13862
PlanLib	-9..617112	5.071738	-1.896	0.06004 .
ClNvaLib	-4.871065	3.436926	-1.417	0.15867
NBI	-4.308106	3.299435	-1.306	0.19384
LaLoma	-2.449653	3.752014	-0.653	0.51492
Palmas	20.965064	11.997125	1.748	0.08279 .
LagLon	23.098280	12.906196	1.790	0.07571 .
RLFlor	25.488093	13.150522	1.938	0.05466 .
AmpLag	-0. 605750	39.532588	-0.015	0.98780
SVL	0.031760	0.023770	1.336	0.18372
Elevation:OrangeBiodom	0.015340	0.006985	2.196	0.02977 *
Elevation:YellowBiodom	0.012341	0.005049	2.444	0.01578 *
Elevation:RedBiodom	-0.006420	0.009644	-0.666	0.50673
Elevation:BlueBiodom	0.011203	0.019228	0.583	0.56111

CT_{min} ~ Elevation + Pop + SVL + BioDom:Elevation				
	Estimate	Std. Error	t value	p-value
Intercept	11.356005	1.414581	8.028	3.7e-13***
Elevation	0.004408	0.006205	0.710	0.47864
GNBII	-1.446772	1.141629	-1.267	0.20717
SatMor	-2.888468	1.572804	-1.837	0.06842 .
NvaLib	-2.517890	4.099168	-0.614	0.54006
Palestina	-9.395028	6.178394	-1.521	0.13063
SalUrb	-9.969761	7.018467	-1.421	0.15770
PlanLib	-6.839257	7.493671	-0.913	0.36300
ClNvaLib	-3.775145	5.084918	-0.742	0.45909
NBI	-4.565826	4.881507	-0.935	0.35124
LaLoma	-8.114239	5.551095	-1.462	0.14607
Palmas	22.058860	17.749743	1.243	0.21604
LagLon	23.636692	19.094713	1.238	0.21785
RLFlor	22.549260	19.456191	1.159	0.24845
AmpLag	11.023658	58.488325	0.188	0.85078
SVL	-0.117657	0.035157	-3.347	0.00105 *
Elevation:OrangeBiodom	0.007213	0.010326	0.699	0.48600
Elevation:YellowBiodom	0.001151	0.007470	0.154	0.87781
Elevation:RedBiodom	-0.019656	0.014268	-1.378	0.17054
Elevation:BlueBiodom	-0.010006	0.028448	-0.352	0.72558

TTR ~ Elevation + Pop + SVL + BioDom: Elevation				
	Value	Std. Error	t value	p-value
Intercept	25.289072	1.859507	13.600	< 2e-16 ***
Elevation	-0.016205	0.008155	-1.987	0.04891 .
GNBII	1.959949	1.500377	1.306	0.19365
SatMor	3.604428	2.067047	1.744	0.08346
NvaLib	7.708967	5.387298	1.431	0.15474
Palestina	4.017297	8.208071	0.489	0.62532
SalUrb	2.702755	9.374068	0.288	0.77354
PlanLib	-2.904955	9.975434	-0.291	0.77133
CINvaLib	-1.087631	6.682841	-0.163	0.87096
NBI	0.263390	6.415496	0.041	0.96731
LaLoma	5.671594	7.295500	0.777	0.43827
Palmas	-1.103364	23.327466	-0.047	0.96234
LagLon	-0.548292	25.095082	-0.022	0.9860
RLFlor	2.926626	25.570157	0.114	0.90905
AmpLag	-11.716451	76.868085	-0.152	0.87908
SVL	0.148825	0.046226	3.219	0.00161
Elevation:OrangeBiodom	0.008270	0.013685	0.604	0.54666
Elevation:YellowBiodom	0.011186	0.009817	1.140	0.25649
Elevation:RedBiodom	0.013245	0.018752	0.706	0.48119
Elevation:BlueBiodom	0.021252	0.037388	0.568	0.57069

Warming Tolerance ~ Elevation + Pop + SVL + BioDom:Elevation				
	Estimate	Std. Error	t value	p-value
Intercept	4.498393	0.942367	4.774	4.59e-06 ***
Elevation	-0.011794	0.004133	-2.854	0.004995 **
GNBII	0.513496	0.809855	0.674	0.501566
SatMor	1.034558	1.047616	0.988	0.325121
NvaLib	9.907296	2.730380	3.629	0.000401 ***
Palestina	-5.778594	4.113144	-1.405	0.162312
SalUrb	-7.461596	4.679445	-1.595	0.113118
PlanLib	-3.922540	4.998034	-0.785	0.433916
CINvaLib	2.096926	3.386980	0.619	0.536870
NBI	4.837432	3.251487	1.488	0.139112
LaLoma	1.253176	3.697488	0.339	0.735184
Palmas	21.975768	11.822779	1.859	0.065206 .
LagLon	24.108993	12.718639	1.896	0.060124 .
RLFlor	33.775888	12.959414	2.606	0.010166 *
AmpLag	13.764359	38.958087	0.353	0.724398
SVL	0.031778	0.023425	1.357	0.177133

Elevation:OrangeBiodom	0.016805	0.006884	2.441	0.015912 *
Elevation:YellowBiodom	0.012341	0.004975	2.480	0.014332 *
Elevation:RedBiodom	-0.006420	0.009504	-0.676	0.500471
Elevation:BlueBiodom	0.011201	0.018949	0.591	0.555403

Cooling tolerance ~ Elevation + Pop + SVL+ Biodom:Elevation				
	Estimate	Std. Error	t value	p-value
Intercept	10.924110	1.405363	7.773	1.54e-12 ***
Elevation	-0.004415	0.006165	-0.716	0.47505
GNBII	1.446061	1.134190	1.275	0.20445
SatMor	2.171463	1.562554	1.390	0.16685
NvaLib	0.801758	4.072455	0.197	0.84421
Palestina	5.822945	6.138132	0.949	0.34444
SalUrb	6.057311	6.972730	0.869	0.38650
PlanLib	1.677172	7.444837	0.225	0.82209
CINvaLib	-0.949350	5.051781	-0.188	0.85121
NBI	0.780392	4.849696	0.161	0.87239
LaLoma	3.019914	5.514920	0.548	0.58485
Palmas	-28.352502	17.634074	-1.608	0.11014
LagLon	-29.931050	18.970279	-1.578	0.11689
RLFlor	-28.240227	19.329401	-1.461	0.14627
AmpLag	-21.150598	58.107176	-0.364	0.71642
SVL	0.116298	0.034928	3.330	0.00111 **
Elevation:OrangeBiodom	-0.007194	0.010258	-0.701	0.48431
Elevation:YellowBiodom	-0.001063	0.007421	-0.143	0.88632
Elevation:RedBiodom	0.019676	0.014175	1.388	0.16733
Elevation:BlueBiodom	0.010106	0.028263	0.358	0.72121

CT_{max} ~ T_{hab_max} + Elevation + Pop + SVL + BioDom:Elevation				
	Estimate	Std. Error	t value	p-value
Intercept	35.274117	88.341623	0.399	0.69030
T_{max_hab}	0.042161	2.751539	0.015	0.98780
Elevation	-0.011794	0.004194	-2.812	0.00565 **
GNBII	0.513487	0.771632	0.665	0.50688
SatMor	0.730780	1.375244	0.531	0.59601
NvaLib	5.389627	13.300854	0.405	0.68596
Palestina	-5.103271	4.318908	-1.182	0.23941
SalUrb	-7.042194	5.156045	-1.366	0.17424
PlanLib	-9.321599	19.906769	-0.468	0.64034
CINvaLib	-4.577275	19.495774	-0.235	0.81473
NBI	-3.922511	25.387866	-0.155	0.87744

LaLoma	-2.293528	10.869670	-0.211	0.83320
Palmas	21.007664	12.304625	1.707	0.09003 .
LagLon	23.140880	13.192290	1.754	0.08165
RLFlor	25.837501	26.275255	0.983	0.32717
AmpLag				
SVL	0.031760	0.023770	1.336	0.18372
Elevation:OrangeBiodom	0.015340	0.006985	2.196	0.02977 *
Elevation:YellowBiodom	0.012341	0.005049	2.444	0.01578 *
Elevation:RedBiodom	-0.006420	0.009644	-0.666	0.50673
Elevation:BlueBiodom	0.011203	0.019228	0.583	0.56111

CT_{min} ~ T_{hab_min} + Elevation + Pop + SVL + BioDom:Elevation				
	Estimate	Std. Error	t value	p-value
Intercept	36.055008	130.949703	0.275	0.78347
T_{max_hab}	-1.110433	5.891633	-0.188	0.85078
Elevation	0.004408	0.006205	0.710	0.47864
GNBII	-1.446772	1.141629	-1.267	0.20717
SatMor	-3.680961	4.489189	-0.820	0.41364
NvaLib	-4.424314	10.956953	-0.404	0.68699
Palestina	-13.346079	21.806569	-0.612	0.54152
SalUrb	-14.297670	23.961182	-0.597	0.55168
PlanLib	-12.554268	31.182541	-0.403	0.68786
ClNvaLib	-8.914123	27.760783	-0.321	0.74861
NBI	-8.672707	22.341227	-0.388	0.69847
LaLoma	-13.648683	29.902405	-0.456	0.64879
Palmas	15.094555	40.931091	0.369	0.71285
LagLon	16.672386	41.530567	0.401	0.68871
RLFlor	16.260905	38.551718	0.422	0.67383
AmpLag				
SVL	-0.117657	0.035157	-3.347	0.00105 **
Elevation:OrangeBiodom	0.007213	0.010326	0.699	0.48600
Elevation:YellowBiodom	0.001151	0.007470	0.154	0.87781
Elevation:RedBiodom	-0.019656	0.014268	-1.378	0.17054
Elevation:BlueBiodom	-0.010006	0.028448	-0.352	0.72558

CT_{max} ~ CT_{min} + Elevation + Pop + SVL + BioDom:Elevation				
	Estimate	Std. Error	t value	p-value
Intercept	38.025016	1.145584	33.193	< 2e-16 ***
CTmin	-0.123486	0.056909	-2.170	0.03177 *
Elevation	-0.011249	0.004159	-2.704	0.00772 **
GNBII	0.334946	0.768256	0.436	0.66355

SatMor	0.361262	1.065068	0.339	0.73499
NvaLib	4.879736	2.746384	1.777	0.07786 .
Palestina	-6.023362	4.209520	-1.431	0.15477
SalUrb	-7.982854	4.802881	-1.662	0.09881 .
PlanLib	-10.169662	5.091751	-1.997	0.04780 *
ClNvaLib	-5.340310	3.409009	-1.567	0.11957
NBI	-4.874019	3.276443	-1.488	0.13919
LaLoma	-3.454240	3.742753	-0.923	0.35770
Palmas	23.692558	11.942113	1.984	0.04929 *
LagLon	26.020730	12.846453	2.026	0.04479 *
RLFlor	28.277122	13.080801	2.162	0.03240 *
AmpLag	0.787727	39.138225	0.020	0.98397
SVL	0.017451	0.024456	0.714	0.47674
Elevation:OrangeBiodom	0.015905	0.006978	2.279	0.02421 *
Elevation:YellowBiodom	0.012485	0.004998	2.498	0.01369 *
Elevation:RedBiodom	-0.008850	0.009612	-0.921	0.35880
Elevation:BlueBiodom	0.009951	0.019043	0.523	0.60213

Table S5. Information of the localities sampled, and microclimatic temperatures recorded in each site of study. The geographic information is in UTM coordinates, elevation in meters. T_{hab_max} is the average daily maximum temperature, T_{hab_min} is the average daily minimum temperature, T_{max} is the maximum temperature recorded, T_{min} is the minimum temperature recorded, T_{mean} is the average of temperature recorded, and days refers to the number of the days of recorded temperature.

Bioclimatic domain	Pop	x	y	Elevation (m)	Datalogger (°C)					
					T_{hab_max}	T_{max}	T_{hab_min}	T_{min}	T_{mean}	Days
Green	GNBII_II	513395	1709839	125						
Green	GNBII	516381	1712152	248	32.13	40.46	22.24	17.87	25.88	734
Green	SatMor	536218	1700986	309	31.81	47.35	21.53	17.94	25.27	560
Green	NvaLib	519484	1713651	768	27.41	34.76	20.53	17.94	22.77	262
Orange	Palest	527176	1747735	736	31.71	47.52	18.68	10.44	23.19	484
Orange	SalUrb	519364	1745037	834	31.39	45.40	18.35	11.03	22.98	480
Orange	PlaLib	508790	1745088	902	25.12	32.98	17.10	8.643	20.77	860
Orange	ArrNeg	494831	1751953	1073						
Yellow	CINLib	521006	1713083	1185	25.16	36.09	17.61	13.86	20.23	352
Yellow	NBI	520347	1719025	1148	22.98	27.58	18.54	13.5	20.28	793
Yellow	LaLoma	538462	1708107	1302	28.43	44.90	17.26	12.85	20.82	814
Red	Palmas	522520	1721465	1401						
Red	LagLon	527235	1722574	1523	31.12	42.48	15.97	11.47	20.61	356
Red	RLFlor	539699	1706913	1494	23.84	38.09	16.58	11.22	19.00	781
Blue	AmpLag	534514	1715118	2137	17.76	23.35	12.31	4.58	14.70	780

Table S6. Mean local humidity in percent per locality sampled during the rainy and dry seasons, as recorded by dataloggers. The number in parentheses represents the minimum and maximum percent recorded.

		Rainy season	Dry season
Bioclimatic domain<br>Pop	Elevation	Mean % Humidity	Mean % Humidity
Green			
GNBII	248	94.19<br>(43.56 – 100)	80.05<br>(28.27 – 100)
SatMor	309	93.84<br>(45.99 – 100)	80.87<br>(27.5 – 100)
NvaLib	768	93.20<br>(54.12 – 100)	70.79<br>(37.27 – 99.43)
Orange			
Pales	736	92.41<br>(43.57 – 100)	75.02<br>(11.09 – 100)
SalUrb	834	91.70<br>(34.42 – 100)	75.42<br>(17.51 – 100)
PlanLib	902	97.75<br>(48.42 – 100)	85.33<br>(22.04) – 100)
Yellow			
CINvaLib	1185	94.85<br>(52.99 – 100)	80.51<br>(36.50 – 100)
NBI	1148	98.40<br>(50.17 – 100)	87.04<br>(44.01 – 100)
LaLoma	1302	93.73<br>(31.71 – 100)	78.84<br>(16.75 – 100)
Red			
LagLon	1523	87.97<br>(39.06 – 100)	77.24<br>(20.48 – 100)
RLFlor	1494	95.56<br>(41.43 – 100)	83.09<br>(27.79 – 100)
Blue			
AmpLag	2137	99.31<br>(67.72 – 100)	93.74<br>(39.16 – 100)

Figures

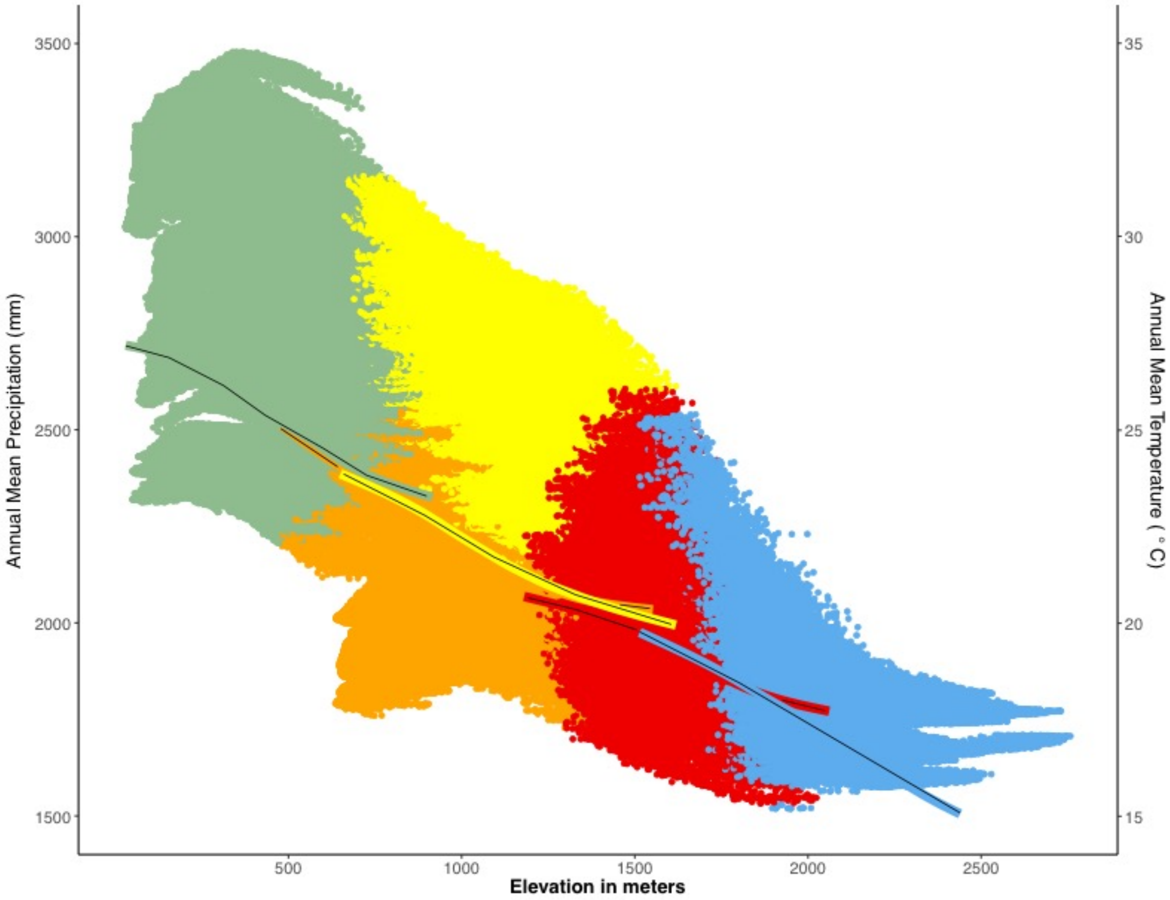

Figure S1. Annual mean precipitation and annual mean temperature (blacklines) along elevational gradient of each bioclimatic domain (BioDom) in the study area (different colors). Green BioDom represent the environment with high annual temperature and precipitation, and lowland areas. Yellow BioDom is characterized by high precipitation in the warmest quarter and intermediate elevations. Orange BioDom characterized by the temperature seasonality and intermediate elevations. Red BioDom with higher elevations from 1180 m – 2061 m a.s.l., and blue BioDom is the area with high precipitation in driest season and high elevations.

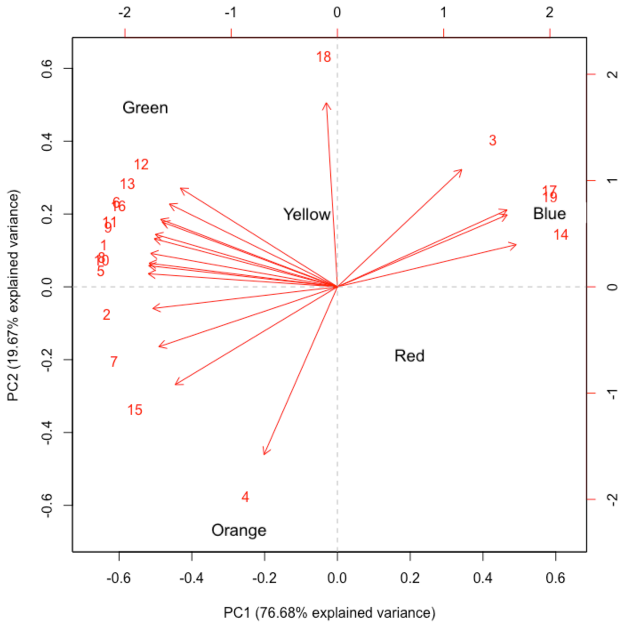

Figure S2. Principal components analysis of the 19 bioclimatic variables for the five bioclimatic domains. Numbers of bioclimatic variables are shown as in Table S1.

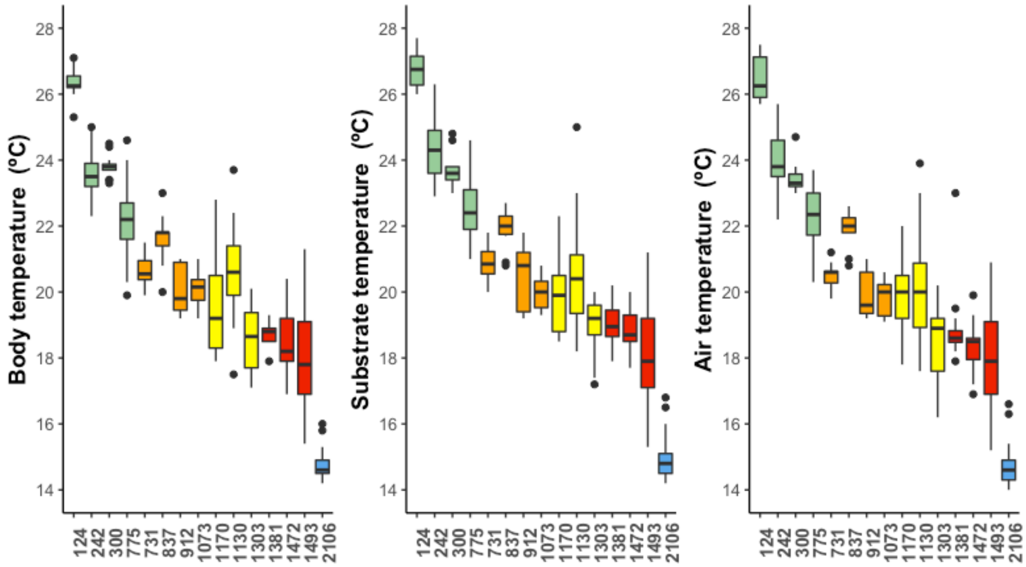

Figure S3. Body temperature (T_b), substrate and air temperature in *Craugastor loki* frogs, along the elevational gradient for different bioclimatic domains (colors) described in Fig. 1 and Table S1. The data were taken only during active hours, from dusk to dawn. The x axis represents the elevation in meters.

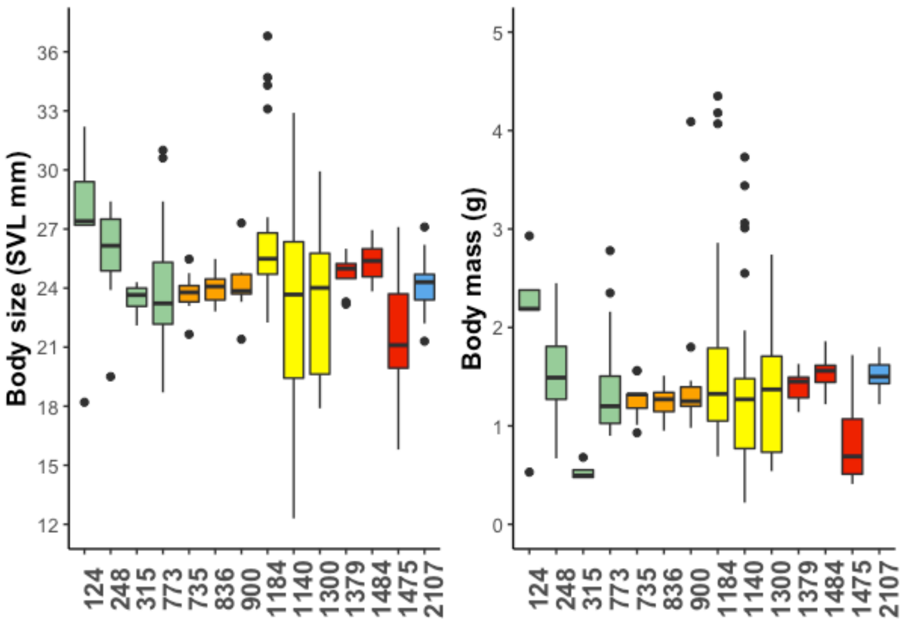

Figure S4. Body size (snout – vent length in mm) and body mass (g) in *Craugastor loki* frogs, along the elevation gradient for different bioclimatic domains (colors) described in Fig. 1 and Table S1. The x axis represents the elevation in meters.

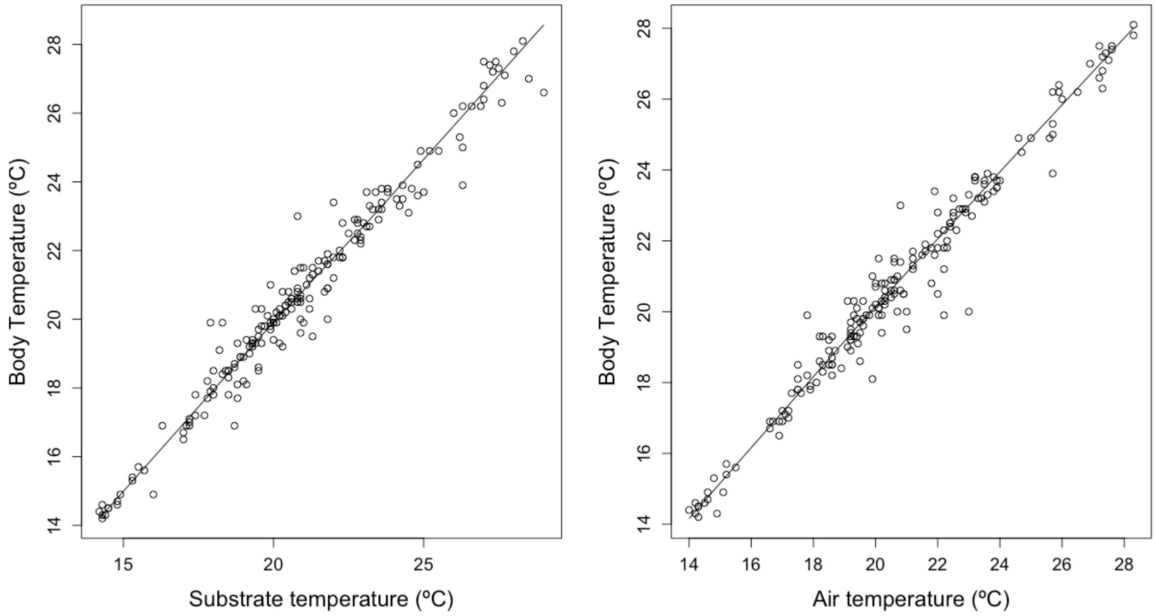

Figure S5. Relationship between body temperature (T_b) and substrate and air temperature of the overall populations sampled (r^2 = 0.9647, p <0.005, n = 191; r^2 = 0.8289, p <0.005, n = 205, respectively.

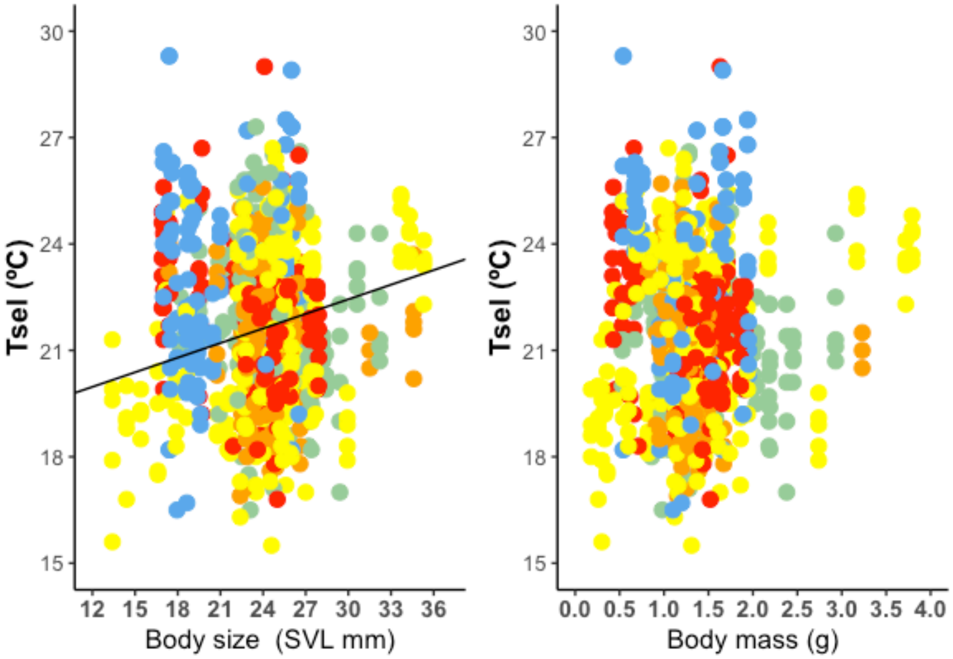

Figure S6. Relationship between T_{sel} with the body size (snout-vent length SVL in mm) (a) and body mass in g (b), along the elevational gradient. Each dot represents one measure of T_{sel} of each one individual. Solid line represents a significant relationship ($p<0.05$). Colors correspond to each bioclimatic domain described in Fig. 1a, Table S1.

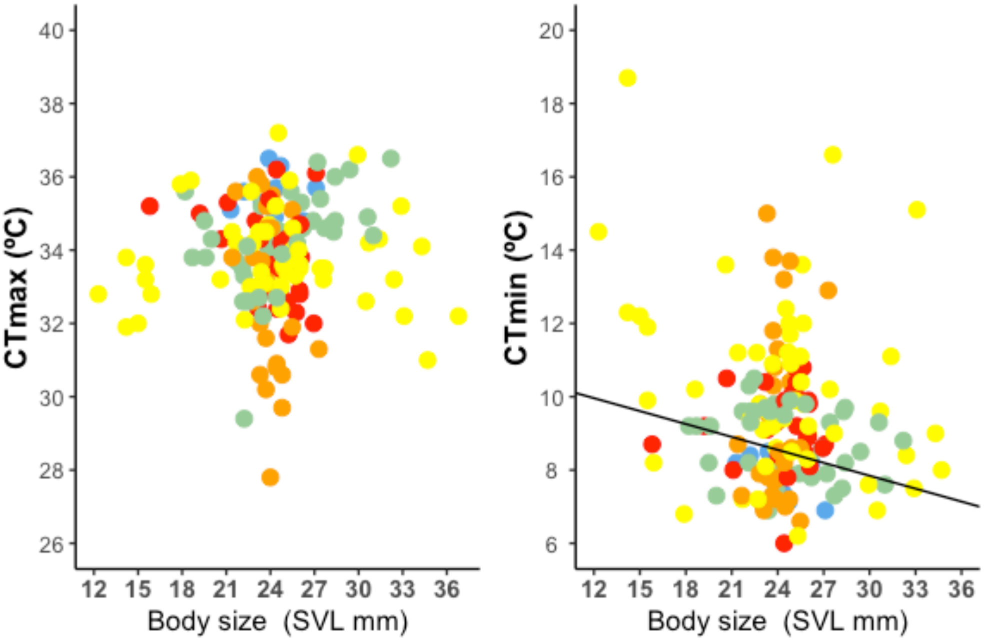

Figure S7. Relationship between CTmax and body size measured as snout-vent length SVL in mm (c) and CTmin with respect to body size (SVL) (d). Solid line represents a

significant relationship ($p<0.05$). Colors correspond to each bioclimatic domain described in Fig. 1a, Table S1.

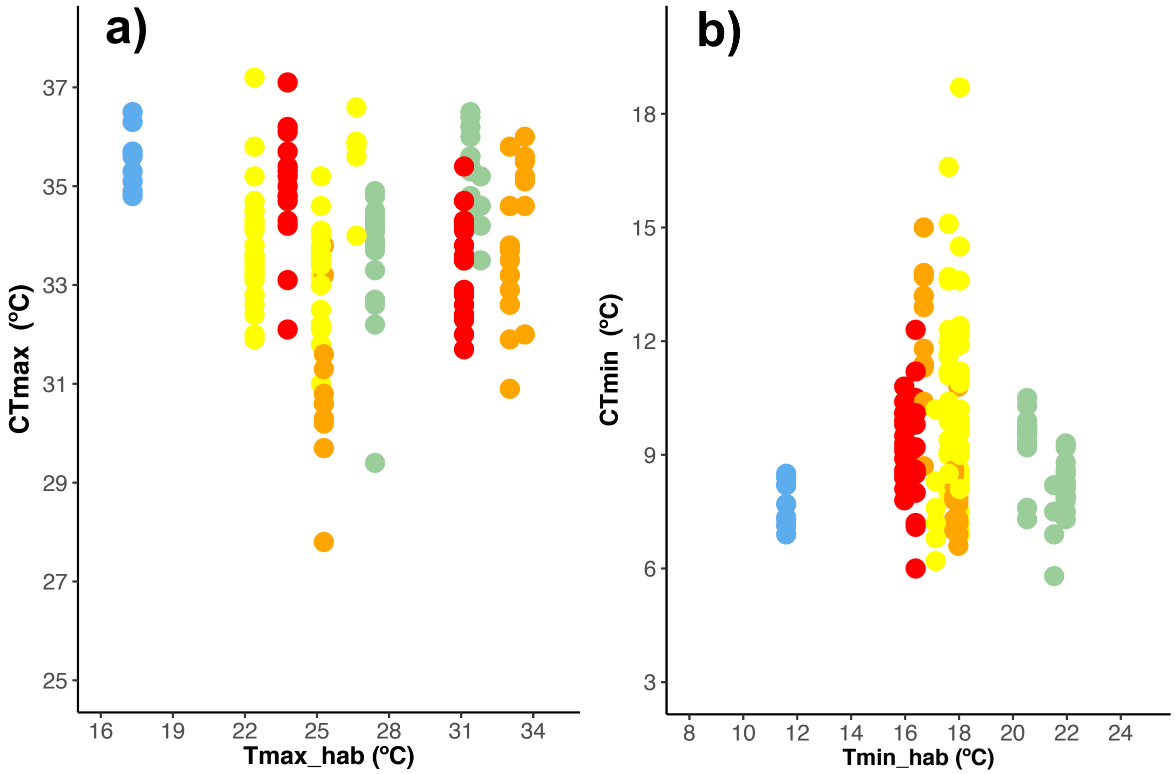

Figure S8. (a) Relationship between critical thermal limits with the maximum daily temperature and (b) minimum daily temperature, along the elevational gradient. Colors correspond to each bioclimatic domain described in Fig. 1a, Table S1.

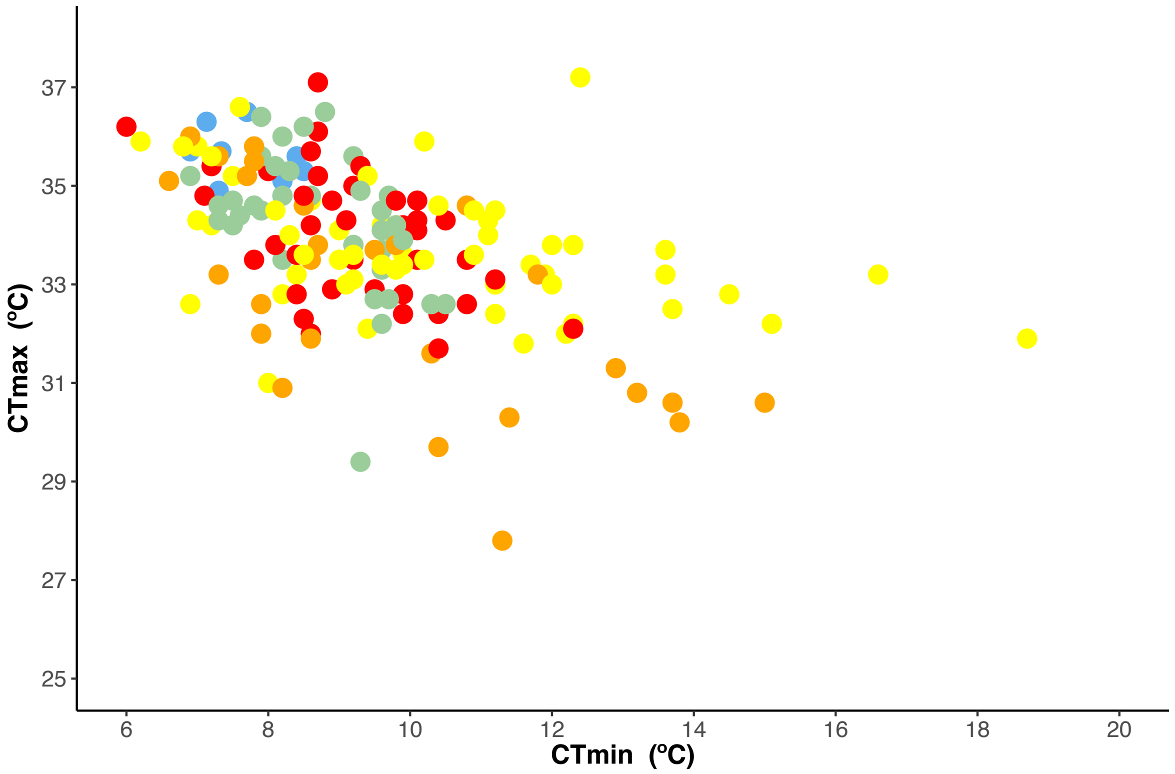

Figure S9. General relationship between both critical thermal limits ($F_{20,135}$ = 10.73, R^2 = 0.556, p<0.0001). Colors represent each bioclimatic domain as described in Fig. 1a, and Table S1.

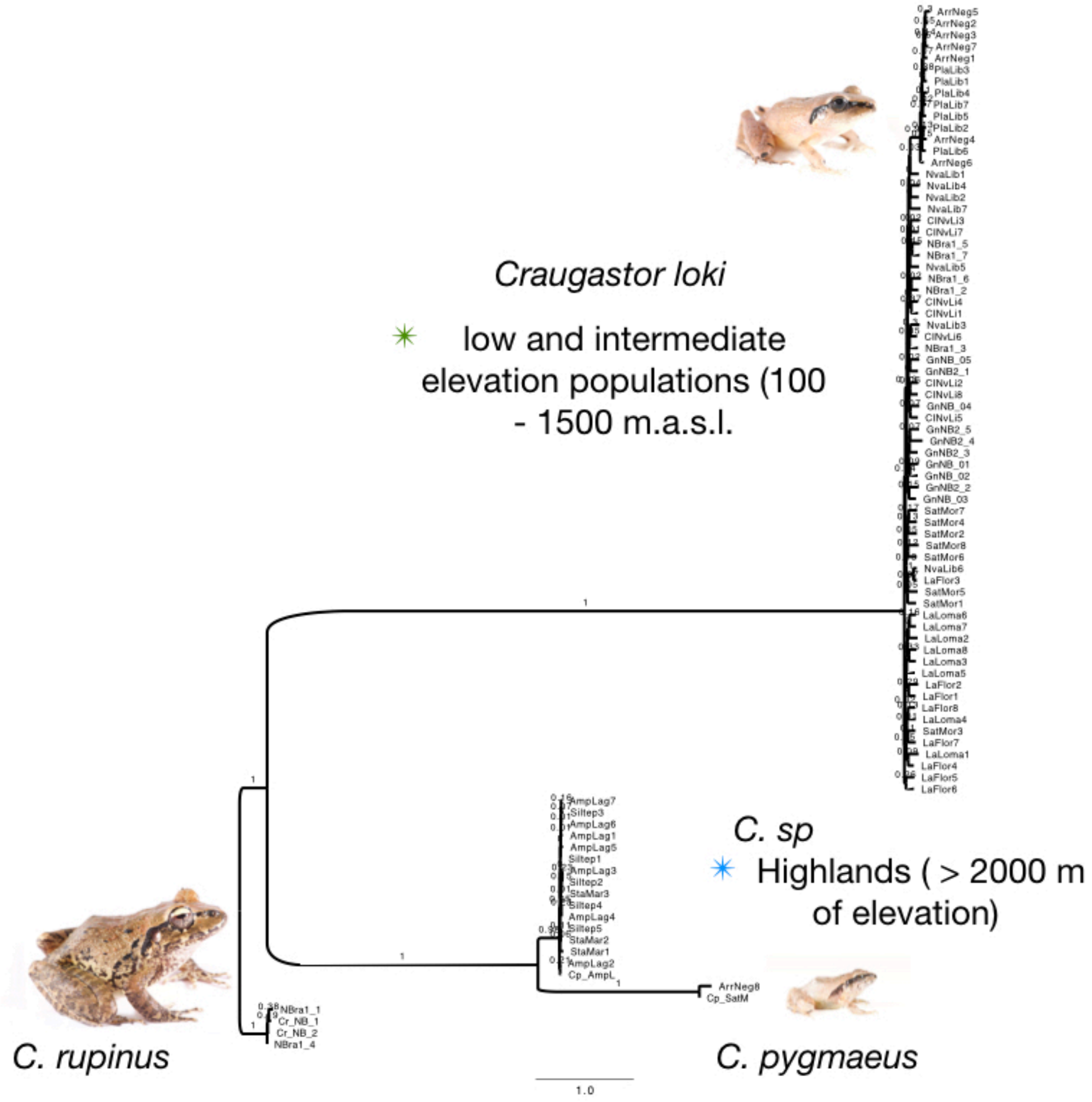

Figure S10. Preliminary results of phylogenetic analysis performed with RADseq data of *Craugastor loki* and outgroups *Craugastor pygmaeus* and *Craugastor rupinus*. Using the maximum-likelihood program RAxML, bootstrap support values are above each node. Individuals from higher elevation (bioclimatic domain blue) represent another species of *Craugastor*. This analysis corresponds of unpublished data on landscape genomics study in preparation.
